# Prototype-oriented contrastive mean-teacher for unsupervised domain adaptive object detection

**DOI:** 10.1038/s41598-026-44991-7

**Published:** 2026-03-27

**Authors:** Qi Cao, Jianwen Tao, Yufang Dan, Di Zhou

**Affiliations:** 1 Institute of Artificial Intelligence Application, Ningbo Polytechnic University, Ningbo, 315800 China; 2 Dazhou City Key Laboratory of Multidimensional Data Perception and Intelligent Information Processing, Sichuan University of Arts and Science, Dazhou, 635000 China; 3 Ningbo Key Laboratory of Aging Health Equipment and Service Technology, Ningbo, 315800 China; 4 Dazhou Industrial Technological Institute of Intelligent Manufacturing, Sichuan University of Arts and Science, Dazhou, China

**Keywords:** Engineering, Mathematics and computing

## Abstract

Unsupervised domain adaptive object detection (UDA-OD) aims to deploy a detector trained on source domain(s) to a new, unlabeled target domain. Carrying out mean-teacher self-training for UDA-OD poses a significant challenge, given that its success depends heavily on the quality of pseudo boxes. While many earlier researches have mainly centered on cross-domain transferability, they often neglect the rich intra- and inter-domain semantic structures. As a result, this neglect empirically restricts the discriminative abilities of the learning model. In our study, we have found a notable alignment and synergy across contrastive learning, prototype learning, and mean-teacher self-training. Building on this insight, we introduce the *P*rototype-*o*riented *C* *o*ntrastive *M*ean *T*eacher (PoCoMT) for UDA-OD, a thorough and flexible framework that seamlessly integrates these three techniques to extract the most beneficial learning signals. Specifically, PoCoMT firstly generate more diverse and reliable probabilistic outputs from self-training through maximizing information entropy and maintaining semantic consistency; secondly, PoCoMT strives to reduce both intra-domain and inter-domain prototypical contrastive learning losses by elaborately designing a Prototype Alignment Network (ProtoAN) module, which fosters intra-domain feature aggregation, aligns inter-domain class structures, and reduces semantic loss between weak and strong augmentations of target domain data. Our ProtoAN can serve as a plugin module for traditional self-training frameworks to tackle the key problem of semantic loss in UDA-OD. Extensive experiments demonstrate that PoCoMT attains new state-of-the-art performance.

## Introduction

Object Detection (OD) has advanced significantly over recent decades due to deep convolutional neural networks, with models like YOLO^1^ and Faster R-CNN^2^ leading the way. However, their performance degrades notably under new environmental conditions (e.g., weather or lighting shifts), a phenomenon called domain shift. To address this, Unsupervised Domain Adaptive (UDA) object detection (UDA-OD) techniques have gained attention^3–9^. Preceding methods aim to transfer pre-trained models from a labeled source domain to an unlabeled target domain with distinct data distributions, using tactics in three main categories: domain alignment, domain translation, and self-training. Domain alignment acquires domain-invariant features via domain classifiers^10^ and gradient reversal layers^11,12^, while domain translation converts labeled source data to match target distributions for adaptation training^13^. Despite accuracy gains from adversarial learning advancements, such as the Fuzzy Inference Attention Module that uses fuzzy logic to model feature transferability and mitigate negative transfer^14^, and the exploration of Partially Transferable Class/ Domain Features (PTCF/PTDF) via rough disentanglement and dynamic adjustment^15^, relying solely on adversarial learning leaves a large performance gap compared to fully supervised Oracle models, highlighting the need for integrated detection models that adapt without bounding box annotations.

To tap into self-training’s untapped potential on unlabeled target domains, researchers have adapted the teacher-student (TS) self-training approach derived from semi-supervised learning for domain adaptation^13,16–26^. This method applies varied augmentations/noise to student and teacher models, uses Exponential Moving Average (EMA) for updates, and enables training via self-supervised learning (e.g., semantic consistency). Recent advances in TS structures include perturbation-agnostic designs that decouple augmentation effects from knowledge transfer^27^, multi-branch trico training that strengthens consistency constraints^28^, Teacher-Student Instance-level Adversarial Augmentation that integrates adversarial perturbations with EMA-stabilized pseudo-labels for domain robustness^29^, and Spatially Enhanced Refined Classifiers that leverage spatial context to dynamically refine pseudo-labels and mitigate noise accumulation^30^. For example, MTOR^18^ adopts the Mean Teacher (MT)^17^ framework, leveraging region-level, inter-graph, and intra-graph consistency to identify relationships. The Unbiased Mean Teacher (UMT)^13^, integrating CycleGAN^21^ into the teacher-student structure, has achieved further gains. Existing approaches have expanded this framework via adversarial learning^22–24^ for cross-domain feature extraction and contrastive learning^25,26,31^ to refine discriminative features, given that Contrastive Learning (CL) can build an approximate domain-invariant feature space^31^. Complementing these advances, prototype-based methods, such as GCN-enhanced prototypes that model relational dependencies to reduce noisy pseudo-label impact^32^, and multi-view collaborative learning that fuses diverse data perspectives^33^ further mitigate intra-domain bias and inter-domain misalignment.

Despite accuracy gains, the cross-domain teacher-student framework faces key challenges. First, teacher-generated pseudo labels often have errors and false positives, even with spatial refinement from spatially enhanced classifiers^30^, noise persists in large domain gaps due to lack of semantic grounding. Second, the MT framework’s intra-domain weak-to-strong augmentation introduces extra semantic shifts/biases between target domain weak and strong features^34^, reducing the reliability of teacher-distilled information, an issue partially addressed by instance-level adversarial augmentation^29^ but not resolved for object-level semantic consistency. Lastly, directly applying contrastive learning^31,35,36^ to cross-domain object detection struggles to identify same-class positive and different-class negative pairs, due to neglecting intra-domain semantic structures^37,38^, a gap highlighted by the feature component transferability analysis in PTCF/PTDF research^15^ but not addressed via category-level alignment.

To address the above limitations, we identify synergies between contrastive learning, prototype learning, and mean-teacher self-training, and propose a novel *P*rototype-*o*riented *Co*ntrastive *M*ean *T*eacher (PoCoMT), which is a holistic framework integrates these three techniques to maximize learning signals. PoCoMT enhances self-training by generating more diverse, reliable probabilistic outputs via boosted information entropy and preserved semantic consistency, building on spatial pseudo-label refinement^30^ and EMA+adversarial stability^29^. It also reduces intra- and inter-domain prototypical contrastive losses through a tailored Prototype Alignment Network (ProtoAN), which promotes intra-domain feature aggregation and aligns inter-domain class structures, integrating prototype-guided enhancement^32^, collaborative learning principles^33^, attention for transferability weighting^14^, and dynamic adjustment of partially transferable features^15^. ProtoAN maps features to a shared space, mitigating semantic biases between domains to ensure consistent class probability predictions and preserve target data’s semantic structure. Instead of domain-specific subnets, it uses class prototypes (learned via contrastive loss) to encode domain infomation, aligning same categories across domains while separating different ones, addressing limitations of standalone TS structures^27,28^, adversarial learning^14,15^, and self-training^30^ via prototype-guided contrastive alignment.

In summary, the core contributions of this paper can be summarized as follows:

- To address the semantic loss problem due to inter-domain as well as intra-domain distribution discrepancies existed in the Mean Teacher (MT) framework for UDA-OD, a corresponding solution is proposed by integrating the core idea of prototype-level contrastive learning. This solution effectively improves the stability of pseudo-labels and the quality of prototypes, providing a new technical approach to solve the key pain points in this field.
- The cross-domain MT framework is extended to construct a Prototype-oriented Contrastive Mean Teacher (PoCoMT) model, which applies prototype-based contrastive learning (CL) to weakly augmented and strongly augmented features to build a shared and unbiased feature space. The core components of this model include: (1) a Mean Teacher self-training module integrated with information maximization, which can generate reliable pseudo-labels robust to strong transformations; (2) an adaptive prototype-focused contrastive learning module, which can reduce inter-domain as well as intra-domain differences and improve the quality of target pseudo-labels.
- A Prototype Alignment Network (ProtoAN) module is designed to effectively bridge the intra-domain and inter-domain semantic gaps by learning a shared feature space. This module applies prototype contrastive learning to weakly/strongly augmented features to achieve the alignment of class prototypes between the source domain and the target domain. It can be flexibly integrated as a plug-in module into mainstream frameworks such as MT and self-supervised learning^39^. Inspired by prototype-guided learning^32^ and multi-view consistency principles^33^, it balances flexibility and practicality.
- Extensive experimental validations are conducted on eight datasets. The results show that the PoCoMT model achieves state-of-the-art performance in the field of UDA-OD. Moreover, when the noise of pseudo-labels increases, it can still maintain performance stability and achieve more significant performance improvement, outperforming the baseline methods that only rely on core UDA strategies or their latest improved versions^14,15,29,30^.

The proposed framework yields three unique advantages tailored to the demands of object detection: (1) noise resilience: MT’s stable pseudo-labels improve prototype estimation, while contrastive alignment refines prototype-feature matching, mitigating cumulative noise; (2) semantic-consistent alignment: prototypes bridge the gap between instance-level CL (lacking semantics) and adversarial methods (loss of discriminability) by aligning features at the category level, critical for preserving object identity across domains; and (3) robustness to domain gap variations: the framework adapts to diverse domain shifts by combining MT’s pseudo-label refinement, prototypes’ semantic grounding, and CL’s feature alignment, integrating complementary strengths of TS structures^27,28^, adversarial learning^14,15^, self-training^30^, and multi-view methods^33^. Experiments on benchmarks confirm that our integration outperforms single-component methods, especially in large domain gap scenarios.

The rest of this paper is structured as follows: “Related work” reviews relevant research (UDA-OD, MT self-training, contrastive learning, prototypical learning). “Methodology” introduces our PoCoMT methodology, with its optimization algorithm in “Prototype-oriented contrastive learning”. “Analysis” analyzes computational complexity, clustering concentration estimation and PoCoMT’s generalization ability. “Experiments” presents and discusses experimental results, and “Conclusion” concludes.

## Related work

This section reviews work related to UDA-OD. To contextualize our work, we organize existing UDA-OD methods into four technical families: unsupervised Domain Adaptation, mean-teacher self-training, prototype-based learning, and contrastive learning, each of which was designed to address domain shift via distinct mechanisms. Below, we synthesize their strengths, limitations, and unmet needs in real-world OD scenarios.

### Unsupervised domain adaptation for object detection

Object detection identifies objects and their positions, with deep learning models (especially anchor-based methods) proving effective. Faster R-CNN^2^, using Region Proposal Networks (RPN) for ROI proposals, is a notable example, followed by other anchor-based studies^40–45^ enhancing performance/efficiency. We use Faster R-CNN as the detection backbone for its adaptability.

To address UDA challenges in object detection, UDA-OD strategies have attracted attention^3–9,34^, aiming to transfer labeled source-trained models to unlabeled target domains with different distributions. UDA-OD is increasingly needed in real-world applications (e.g., self-driving, edge AI) where domain shifts are common and labeled target data is costly.

Recent UDA-OD progress, with source data access, includes five tactics: adversarial feature learning^3,5,46–48^ (using gradient reversal layers like DANN^11^); pseudo-labeling^49–51^ (training on high-confidence target predictions); image-to-image translation^3,8,52,53^ (unpaired translation for cross-domain conversion); domain randomization^53,54^ (stylized source data for robust training); and Mean-Teacher training^13,18^ (incremental unlabeled data training for generalization). For example, adversarial methods^3,5,7,48^ use domain discriminators to learn domain-invariant features; translation methods^8,49,52^ synthesize cross-domain images to reduce gaps. Adversarial learning has evolved beyond basic discriminators, with fuzzy inference attention modules that adaptively weight domain-invariant features to mitigate negative transfer^14^, and PTCF/PTDF-based methods that avoid strict feature separation and dynamically utilize latent transferable information^15^. Self-training advances include spatially enhanced classifiers that refine pseudo-labels via spatial context^30^, while Mean-Teacher has been enhanced with instance-level adversarial augmentation to boost domain robustness^29^.

### Mean teacher self-training

Self-training, using teacher-student reciprocal learning to improve unlabeled target performance^17–20^, has grown prominent. It enables models to generate pseudo-labels for unlabeled targets, avoiding supplementary methods (e.g., adversarial learning) and showing promise, as in the STAC framework for semi-supervised OD^55^. However, domain shift can lead to incorrect target pseudo-labels, degrading performance.^51^ reduced noisy pseudo-labels by modeling proposal distribution, but our architecture-agnostic approach works with single-stage detectors.^49^ combined domain transfer with pseudo-labeling, also architecture-agnostic.

Mean Teacher^17^ was extended from semi-supervised OD to UDA-OD by^18^. Subsequent advances include: Unbiased Mean Teacher (UMT)^13^ (integrating image translation); MTOR^18^ (region-level and graph-structural consistencies via extra regularization); Adaptive Teacher^19^ (weak-strong augmentation with adversarial training); and Probabilistic Teacher (PT)^20^ (uncertainty-guided pseudo-labeling for classification/localization). Despite leading UDA-OD, low-quality teacher-generated pseudo-labels remain a key hurdle^56^.

Recent innovations in student-teacher structures have targeted these limitations, including perturbation-agnostic designs that decouple augmentation effects from knowledge transfer^27^, multi-branch trico training that introduces complementary supervision signals^28^, instance-level adversarial augmentation that enhances domain robustness while maintaining EMA stability^29^, and spatial context-enhanced classifiers that refine pseudo-label quality^30^. While these methods improve TS robustness and transfer efficiency, they lack integration with prototype-based and contrastive learning to address inter-domain misalignment, a gap our framework fills by synthesizing their complementary strengths.

### Contrastive learning

Contrastive learning, for unsupervised representation learning^57^, draws similar (positive) pairs close and pushes dissimilar (negative) pairs apart in feature space. It has driven self-supervised visual pre-training, aided by large batches^39^, memory banks^58^, asymmetric architectures^59^, or clustering^60^, surpassing supervised pre-training in some cases^61^.

To align with downstream tasks beyond image classification (e.g., OD, semantic segmentation), detailed approaches use masks^62,63^, objects^64^, or regions^65^. Our prototype-level contrastive learning, inspired by this, enhances domain-adaptive detectors via noisy pseudo-labels and prototype-level contrast, differing from standard feature-level methods by using predicted classes from pseudo-labels to build pairs and optimize object-level features. Contrastive learning in teacher-student detection frameworks^66,67^ has been explored, but ours is the first to analyze synergy between Mean Teacher^17^ (and its adversarial augmentation advance^29^), prototypical learning, and contrastive learning, integrating insights from perturbation-agnostic TS^27^, trico training^28^, spatial pseudo-label calibration^30^, and adversarial transferability weighting^14,15^.

### Prototype-based learning

Prototype-based learning works in unsupervised domain adaptation^68–72^, calculating prototypes by averaging target pseudo-label features. It appears across contexts: open-world OD (class separation, unknown class identification^73^); semi-supervised OD (class distribution alignment^74^); cross-domain OD (foreground/background alignment^75^); few-shot OD (universal prototypes for invariant characteristics^76^). In contrast, we apply prototypes in UDA-OD to streamline domain-specific feature learning.

Robust clustering requires well-separated prototypes/clusters. ProtoNCE^72^ learns single-domain semantic structure via iterative clustering/representation learning, grouping same-cluster features and separating different ones. However, direct application in domain adaptation may miscluster: distinct classes from different domains into the same cluster, or same classes from different domains into distant clusters, due to domain shift.

A key recent advancement in prototype-based domain adaptation is the use of GCN to enhance prototype quality^32^, capturing relational dependencies between samples to reduce sensitivity to noisy pseudo-labels, critical for UDA-OD where proposal-level noise degrades prototype reliability. This approach addresses limitations of traditional prototype averaging but has not been integrated with Mean-Teacher adversarial augmentation^29^, self-training spatial refinement^30^, or adversarial transferability optimization^14,15^ to jointly optimize intra- and inter-domain alignment. Our ProtoAN module builds on this idea, combining prototype-guided refinement with contrastive learning to align source and target prototypes effectively while leveraging these core UDA strategy advances.

### Limitations of alternative tactics

Standalone Mean-Teacher (MT) leverages EMA of student model weights to generate stable pseudo-labels, addressing label scarcity in UDA. However, in OD, pseudo-labels are prone to noise due to domain shift, even with adversarial augmentation^29^, perturbation-agnostic designs^27^, and trico training^28^, MT lacks prototype-guided semantic alignment, leading to accumulated errors.

Vanilla Contrastive Learning (CL) fails to encode category-level semantic structures, and is sensitive to outliers. Multi-view collaborative learning^33^ mitigates this but does not address category-level alignment gaps.

Prototypes effectively capture category semantics but suffer from noisy estimation in UDA-OD. GCN-enhanced prototypes^32^ improve reliability but lack integration with TS and CL, while standalone adversarial learning, even with fuzzy inference attention^14^ and partial feature disentanglement^15^, struggles with semantic discriminability. Standalone self-training^30^ improves pseudo-label calibration but lacks stability and semantic grounding.

Our framework integrates the three components to address these limitations, leveraging their complementary strengths for UDA-OD:: (1) the MT module generates high-confidence pseudo-labels for target objects by smoothing student model predictions via EMA; (2) prototypes (computed as class-level feature centroids from labeled source data and high-confidence target pseudo-labels) provide a compact representation of category-specific features; and (3) contrastive alignment addresses two key gaps: First, it reduces prototype noise by iteratively pulling object features toward their class prototypes, enhancing prototype quality; Second, it avoids over-alignment by preserving target domain feature characteristics (via asymmetric alignment), a critical advantage for OD where target-specific object layouts must be retained. This combination ensures that features are both domain-invariant and category-discriminative, thus solving the core tradeoff for UDA-OD.

**Fig. 1:**
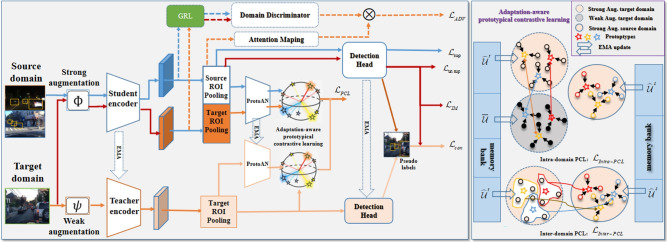
The workflow of PoCoMT (optimally viewed in color) comprises two primary modules (Solid lines represent the core workflow of the PoCoMT framework; Dashed lines denote the optional integration of adversarial learning into the framework, which aligns the feature distributions between two domains): (1) Cross-domain mutual learning with information maximization (left). To generate exact and reliable pseudo labels for target domain images, we supply images with weak augmentation as input to the Teacher (to deliver trustworthy pseudo-labels) whereas images with strong augmentation serve as inputs for the Student. The Student model is trained via standard gradient updates, while the Teacher model undergoes updates using the exponential moving average (EMA) of the Student’s weights. We also integrate the Information Maximization (IM) loss to ensure that the prediction output of target features displays both individual certainty and global variety. (2) Adaptation-aware prototypical contrastive learning (Right). We propose minimizing prototypical contrastive losses for object-level representation learning to boost the performance of mean teacher self-training through a carefully designed plug-in module, ProtoAN. This module extracts compact ROI feature representations for each proposal.

## Methodology

### Problem statement

In the scenario of UDA-OD, the labeled source domain is denoted as $\mathcal {D}_s = \{(I_i^s, Y_i^s)\}_{i=1}^{N_s}$, where: $I_i^s$ represents the $i$th image in the source domain; $Y_i^s = \{(b_{i,j}^s, c_{i,j}^s)\}_{j=1}^{M_{s,i}}$ denotes the annotation of $I_i^s$, including bounding boxes and corresponding object classes for all objects in $I_i^s$; $b_{i,j}^s$ is the bounding box coordinate of the $j$-th object in the $i$-th source image (formatted as $[x_{\text {min}}, y_{\text {min}}, x_{\text {max}}, y_{\text {max}}]$ to define the object’s spatial location); $c_{i,j}^s \in \mathcal {C}$ is the category label of the $j$-th object in the $i$-th source image, where $\mathcal {C} = \{1, 2, \dots , C\}$ denotes the set of all object categories shared by the source and target domains; $M_{s,i}$ is the number of objects contained in the $i$-th source image; $N_s$ is the total number of images in the source domain. The unlabeled target domain is defined as $\mathcal {D}_t = \{(I_i^t, Y_i^t)\}_{i=1}^{N_t}$, where $I_i^t$ represents the $i$-th image in the target domain; $Y_i^t = \{(b_{i,j}^t, c_{i,j}^t)\}_{j=1}^{M_{t,i}}$ denotes the unobserved annotation of $I_i^t$ (consistent with the annotation format of $Y_i^s$), which is unknown during model training and can be initialized randomly for iterative optimization; $b_{i,j}^t$ and $c_{i,j}^t$ correspond to the bounding box and category label of the $j$-th object in the $i$-th target image, respectively;$M_{t,i}$ is the number of objects contained in the $i$-th target image; $N_t$ is the total number of images in the target domain; All target samples $\{I_i^t\}_{i=1}^{N_t}$ follow an identical target domain distribution $\mathcal {P}_t$, which is distinct from the source domain distribution $\mathcal {P}_s$ of $\{I_i^s\}_{i=1}^{N_s}$ (i.e., $\mathcal {P}_s \ne \mathcal {P}_t$), resulting in domain shift. For the convenience of description, we denote the concatenated set of all source and target images as $\mathcal {I} = [\mathcal {I}^s; \mathcal {I}^t]$, where $\mathcal {I}^s = \{I_i^s\}_{i=1}^{N_s}$ and $\mathcal {I}^t = \{I_i^t\}_{i=1}^{N_t}$; the concatenated set of all annotations is $\mathcal {Y} = [\mathcal {Y}^s; \mathcal {Y}^t]$, where $\mathcal {Y}^s = \{Y_i^s\}_{i=1}^{N_s}$ and $\mathcal {Y}^t = \{Y_i^t\}_{i=1}^{N_t}$ (note that $\mathcal {Y}^t$ remains unobserved).

The primary goal of UDA-OD is to leverage the labeled source domain $\mathcal {D}_s$ and unlabeled target domain $\mathcal {D}_t$ to construct a domain-invariant object detector, which can effectively predict accurate bounding boxes and category labels for objects in target images despite the domain shift between $\mathcal {P}_s$ and $\mathcal {P}_t$.

### Overall formulation

Self-training relies on generated pseudo labels, which may contain noise. This stems from two factors: intra-domain divergence between weakly and strongly augmented data^34^, and the inter-domain gap separating source and target datasets during training. As a result, the standard mean-teacher model^19^ might fail to reach optimal performance. Building on the teacher-student framework^19,20^, our PoCoMT approach comprises two key parts: mean teacher self-training with information maximization through a Teacher-Student (TS) model, and adaptation-aware prototypical contrastive learning via a carefully crafted Prototype Alignment Network (ProtoAN) module. The detector structures in both branches of the TS model are rooted in the Faster-RCNN architecture^2^. Acting as a plug-in component, ProtoAN acquires a shared space that lessens the bias between weak and strong features. Our PoCoMT extracts lossless knowledge from weak features, while contrastive learning applied to strong features encourages the extraction of cross-domain representations. The overall architecture and workflow of the proposed PoCoMT framework are illustrated in Fig.  1, with distinct line notations to distinguish core processes and optional components. Solid lines depict the framework’s essential workflow, which consists of two interrelated modules: cross-domain mutual learning with information maximization (left part) and adaptation-aware prototypical contrastive learning (right part). Dashed lines in the figure represent an optional extension: the integration of adversarial learning into the MT framework, which can be added to further align source and target domain feature distributions and complement the core self-training and prototype alignment mechanisms. This optional design ensures the framework’s flexibility to adapt to different domain shift scenarios.

The training pipeline of PoCoMT is illustrated in Fig. 1 , which mainly encompasses four stages: (1) Pretraining. The detector is trained using labeled source data for initialization, after which the trained weights are copied to both the teacher and student models. (2) Cross-domain mutual learning with information maximization^77^. This stage ensures that the prediction results of target features display both individual certainty and global variety. (3) Domain-invariant adversarial learning with attention. This optional module aims to align distributions across the two domains, a step that can further lower the false positive rate in pseudo label generation. (4) Adaptation-aware prototypical contrastive learning. To further reduce both intra-domain and inter-domain biases, we suggest minimizing prototypical contrastive losses for object-level representation learning. This is achieved by elaborately designing a ProtoAN module that extracts compact ROI feature representations for each proposal.

Our objective loss expands the Mean-Teacher loss by incorporating Information Maximization for cross-domain object detection, referred to as $\mathcal {L}_\mathrm{{mt-im}}$ including mean-teacher self-training loss $\mathcal {L}_{mt}$ and information maximization loss $\mathcal {L}_{IM}$, and integrating the adaptation-aware prototypical contrastive loss (PCL) $\mathcal {L}_\mathrm{{pcl}}$. This PCL includes both intra-domain PCL $\mathcal {L}_{\mathrm {Intra-PCL}}$ and inter-domain PCL $\mathcal {L}_{\mathrm {Inter-PCL}}$. The complete objective of PoCoMT is expressed through Eq. (1).

1 $$\begin{aligned} \begin{aligned} \mathcal {L}_\mathrm{{PoCoMT}}&= \min _{\varTheta } \mathcal {L}_{mt-im} +\lambda \mathcal {L}_{pcl}, \\ \textrm{where},\\ \mathcal {L}_{{mt}-im}&=\mathcal {L}_{mt}+\mathcal {L}_{IM},\quad \mathcal {L}_{{pcl}}&=\mathcal {L}_{\mathrm {Intra-PCL}}+\mathcal {L}_{\mathrm {Inter-PCL}}, \end{aligned} \end{aligned}$$

where $\lambda$ is a trade-off hyper-parameter between MT self-training and prototypical contrastive learning, and $\varTheta = \{\varTheta _{{mt-im}}, \varTheta _{{{ProtoAN}}}\}$ are parameters of models from the strong branch, in which $\varTheta _{{mt-im}}=\{\varTheta _{{wa}},\varTheta _{{sa}}\}$ stands for the weak and strong branches parameters, respectively. During the model training, we optimize the strong branch iteration-wise, while the EMA updating on the weak branch is triggered epoch-wise. The concrete training is presented in Algorithm 1. In the following subsections, we will design the content of each part respectively.

### Cross-domain mean-teacher with information maximization

The standard mean teacher (MT) approach utilizes a self-supervised training mechanism. In particular, within the strong branch, the heavily augmented image $\hat{\boldsymbol{I}}$ derived from the original image $\boldsymbol{I}$ is first transformed into image features through a deep neural network. It is then refined into M strong (RoI) features $\hat{\boldsymbol{X}}=\{\hat{\boldsymbol{x}}_i\}_{i=1}^M$, which are tailored to teacher-generated proposals produced by the RPN in the weak branch. Subsequently, the RCNN module generates predictions $\hat{Y}=\{\hat{b}_i,\hat{c}_i\}_{i=1}^{M}$ for these features. Here, $\hat{b_i}$ and $\hat{c}_i$ stand for the bounding boxes and category distributions corresponding to the i-th instance in $\hat{\boldsymbol{I}}$. When the target image $\bar{\boldsymbol{I}}$ with weak augmentation goes through the weak branch, we acquire M weak (RoI) features $\bar{\boldsymbol{X}}=\{\bar{\boldsymbol{x}}_i\}_{i=1}^{M}$ along with their associated predictions $\bar{Y}=\{\bar{b}_i,\bar{c}_i\}_{i=1}^{M}$. Let $\bar{Y}^h=\{\bar{b}_i^h,\bar{c}_i^h\}_{i=1}^{M}$ denote the high-confidence instance predictions within $\bar{Y}$. The objective of MT is defined as follows:

2 $$\begin{aligned} \begin{aligned} \mathcal {L}_{mt}&= \mathcal {L}_{det}(\hat{I}, \bar{Y}^h) + \mathcal {L}_{con}(\hat{I},\bar{Y}), \\ \textrm{where,}\\ \mathcal {L}_{det}(\hat{I}, \bar{Y}^h)&= \mathcal {L}_{rpn}(\hat{I}, \bar{b}^h) + \mathcal {L}_{rcnn}(\hat{I}, \bar{\boldsymbol{a}}^h),\quad \mathcal {L}_{con}(\hat{I}, \bar{Y})&= \frac{1}{M}\sum D_\mathrm{{KL}}(\hat{c}_i||\bar{c}_i), \end{aligned} \end{aligned}$$

where $\sum D_\mathrm{{KL}}$ is the Kullback–Leibler divergence function, $\bar{\boldsymbol{a}}^h$ is the one-hot version of $\bar{c}^h$, and $\mathcal {L}_{det}$ derives from the Fater-RCNN paradigm, consisting of location regression term ($\mathcal {L}_{rpn}$) and classification term ($\mathcal {L}_{rcnn}$), $\mathcal {L}_{con}$ presents a semantic consistency regularization, which computes a pseudo label for an unlabeled sample from its weakly-augmented version and applies the pseudo label on its strongly augmented version for the cross-entropy loss optimization.

In cross-domain learning scenarios, knowledge is transferred reciprocally between the Teacher and Student models, though the forms of transfer differ between the two directions. Below, we outline the mutual learning process within our proposed framework.

#### Mutual learning between teacher and student

Self-training is critical to the MT framework, as the teacher generates reliable pseudo-labels for the unannotated target domain to optimize the student. We first use supervised source data $\mathcal {D}_s = \{({I}_i^s, {Y}_i^s)\}$ with supervised loss $\mathcal {L}_{sup}$ to train and initialize the student:

3 $$\begin{aligned} \begin{aligned} \mathcal {L}_{sup}({\hat{X}}_s, {\hat{B}}_s, \hat{C}_s) = \mathcal {L}_{cls}^{rpn}(\hat{X}_s, {\hat{B}}_s, \hat{C}_s)+\mathcal {L}_{reg}^{rpn}({X}_s, \hat{B}_s, \hat{C}_s) +\mathcal {L}_{cls}^{roi}({X}_s, \hat{B}_s, \hat{C}_s)+\mathcal {L}_{reg}^{roi}({X}_s, \hat{B}_s, \hat{C}_s), \end{aligned} \end{aligned}$$

where RPN loss $\mathcal {L}^{rpn}$ (for proposal generation) and ROI loss $\mathcal {L}^{roi}$ (for ROI prediction) both include classification (cls) and regression (reg). Binary cross-entropy loss is used for $\mathcal {L}_{cls}^{rpn}$ and $\mathcal {L}_{cls}^{roi}$, with $l_1$ loss for $\mathcal {L}_{reg}^{rpn}$ and $\mathcal {L}_{reg}^{roi}$.

Knowledge transfer from student to teacher: The student is updated via gradient descent to minimize detection loss, while the teacher’s weights $\theta ^T$ are updated as the exponential moving average (EMA) of the student’s weights $\theta ^S$:

4 $$\begin{aligned} \theta ^T \leftarrow \rho \theta ^T + (1-\rho )\theta ^S, \end{aligned}$$

where $\rho \in [0,1)$ (0.9996 in our setup) ensures smooth updates. The teacher, an ensemble of historical students, provides stable targets and is used for evaluation.

Knowledge transfer from teacher to student: The teacher detects target-domain objects, generates pseudo-labels via post-processing (e.g., confidence filtering, non-maximum suppression), and transfers knowledge by aligning the student’s predictions with these pseudo-labels. The student is updated using:

5 $$\begin{aligned} \begin{aligned} \mathcal {L}_{unsup}(\hat{X}_t, \hat{{C}}_t) = \mathcal {L}_{cls}^{rpn}(\hat{X}_t, \hat{{C}}_t) +\mathcal {L}_{cls}^{roi}(\hat{X}_t, \hat{{C}}_t), \end{aligned} \end{aligned}$$

where $\hat{\mathcal {C}}_t$ denotes teacher-generated target pseudo-labels. Unsupervised losses exclude bounding box regression, as unlabeled data’s bounding box confidence reflects only category certainty, not position accuracy.

#### Information maximization

We further incorporate the information maximization (IM) loss^77^ to guarantee that the prediction output of target features exhibits both individual certainty and global diversity. Specifically, we jointly minimize the entropy $\mathcal {L}_{{ent}}$ and maximize the diversity $\mathcal {L}_{{div}}$ to constitute the IM loss ($\gamma =1$):

6 $$\begin{aligned} \begin{array}{l} \begin{aligned} \mathcal {L}_{IM}(\hat{I}, \bar{Y}) & =\mathcal {L}_{{ent }}(\hat{I}, \bar{Y})+\mathcal {L}_{{div }}(\hat{I}, \bar{Y}), \\ \textrm{where,}\\ \mathcal {L}_{{ent }}(\hat{I}, \bar{Y}) & =-\mathbb {E}_{x_j \in \hat{I}} \sum _{k=1}^{K} \hat{c}_k^j \log \hat{c}_k^j, \quad \mathcal {L}_{{div }}(\hat{I}, \bar{Y}) & =D_{K L}(\hat{c}, \frac{1}{K} {\bf 1}_{K})-\log K, \end{aligned} \end{array} \end{aligned}$$

where ${\bf 1}_{K}\in R^K$ is a all-ones vector, and $\hat{c}=\mathbb {E}_{x_j \in \mathcal {\hat{I}}}\left[ \hat{c}_j\right]$ represents the average output over the entire strongly augmented data.

The introduced IM loss exhibits potential to outperform the conditional entropy minimization, a technique frequently utilized in preceding UDA methodologies. This superiority arises from its ability by incorporating the diversification loss $\mathcal {L}_{{div}}$ to surpass the trivial solution, wherein all target data approach to the similar or even the same one-hot encoding. Additionally, to minimize the entropy loss $\mathcal {L}_{ent}$, the target data would be adjusted to move closer to a certain one-hot code.

#### Adversarial learning to bridge domain bias

Since the Student processes images from both domains, adversarial loss^78^ can be applied to it for distribution alignment. For implementation, a domain discriminator $D$ is placed after the feature encoder $E$ in the Student model (Fig. 1), tasked with identifying whether the extracted feature $E(\hat{I})$ comes from the source or target domain. We define $D(E(\hat{I}))$ as the probability of an input sample belonging to the target domain, and $1 - D(E(\hat{I}))$ as that of belonging to the source domain. The discriminator $D$ is updated via binary cross-entropy loss, with input images assigned domain labels $y_d$: $y_d=0$ for source-domain images and $y_d=1$ for target-domain images. The discriminator loss $\mathcal {L}_{dis}$ is formulated as:

7 $$\begin{aligned} \begin{aligned} \mathcal {L}_{dis} = - y_d \log D(E(\hat{I})) - (1-y_d) \log (1-D(E(\hat{I}))). \end{aligned} \end{aligned}$$

On the other hand, the feature encoder *E* is encouraged to produce features that confuse the discriminator *D* while the discriminator *D* aim to distinguish which domain the derived features are from. Hence, such adversarial optimization objective function can be defined as the following:

8 $$\begin{aligned} \begin{aligned} \mathcal {L}_{adv} = \max _{E} \min _{D} \mathcal {L}_{dis}. \end{aligned} \end{aligned}$$

Note that object detection tasks require both localization and classification of objects, with RoIs generally being more important than background regions. However, domain classifiers align all spatial positions of the entire image without focus, which may degrade adaptation performance. To solve this problem, we further propose an attention mechanism^79^ to achieve foreground-aware distribution alignment. Specifically, given an image $x\in \hat{I}$ from any domain, we denote $F_{rpn}(x) \in \mathbb {R}^{H \times W \times C}$ as the output feature map of the convolutional layer in the RPN module, where $H \times W$ and C are the spatial dimensions and the number of channels of the feature map, respectively. Then, we construct a spatial attention map by averaging activation values across the channel dimension. Moreover, we filter out (set to zero) values smaller than a given threshold, which are more likely to belong to background regions. The attention map $A(x) \in \mathbb {R}^{H \times W}$ is formulated as:

9 $$\begin{aligned} \begin{aligned} M(x) = S(\frac{1}{C}\sum \limits _{c} | F_{rpn}^c(x) | ),\\ T(x) = \frac{1}{HW}\sum \limits _{h,w} M(x)^{(h, w)},\\ A(x) = 1\!\!1(M(x) > T(x)) \otimes M(x) , \end{aligned} \end{aligned}$$

where $1\!\!1$ is an indicator function, *M*(*x*) stands for the attention map before filtering, and $S(\cdot )$ is the sigmoid function. $F_{rpn}^c(x)$ represents the *c*-th channel of the feature map. $\otimes$ denotes the element-wise multiplication. Threshold *T*(*x*) is set to the mean value of *M*(*x*).

Therefore, the total objective of the domain adversarial learning module is defined as:

10 $$\begin{aligned} \begin{aligned} \mathcal {L}_{ADV} = \sum \limits _{h,w} (1 + A(x)^{(h,w)}) \cdot \mathcal {L}^{h,w}_{dis}\quad , \end{aligned} \end{aligned}$$

where $\mathcal {L}^{h,w}_{dis}$ stands for the adversarial loss on pixel (*h*, *w*). Combining adversarial learning with the attention mechanism, the domain adversarial learning module aligns the feature distributions of foreground regions that are more transferable for the detection task.

In a nutshell, our Cross-domain Mean-Teacher with Information Maximization loss is defined by incoperating Eqs. (3), (5), $\mathcal {L}_{con}$ in Eqs. (2), and (6):

11 $$\begin{aligned} \begin{aligned} \mathcal {L}_{mt-im}=\underbrace{\mathcal {L}_{sup}+\mathcal {L}_{unsup}+\mathcal {L}_{con}}_{\mathcal {L}_{mt}}+\mathcal {L}_{IM}+\beta \mathcal {L}_{ADV}, \end{aligned} \end{aligned}$$

where $\beta$ is a trade-off hyper-parameter.

### Prototype-oriented contrastive learning

Although creating distinct disparities, the intra-domain weak-to-strong augmentation in cross-domain MT framework (Eq. (11)) introduces extra semantic shifts/biases between target domain’s weak and strong features, undermining the reliability of distilled information from weak features. Moreover, pseudo-labels from MT predictions are untrustworthy under distribution shift, hindering direct use of much valuable information.

In summary, pseudo-labels for self-training may be noisy due to intra-domain shift (weak vs. strong augmentations) and inter-domain gap (source vs. target datasets), leading to sub-optimal MT models. To address this, we propose prototypical contrastive learning for object-level representation learning to enhance mean teacher self-training. Specifically, we designed a Prototype Alignment Network (ProtoAN) to extract compact ROI features for each proposal. The target intra-domain prototype mining module searches the weakly augmented embedding space and supervises strongly augmented embeddings via prototypical contrastive learning. Meanwhile, inter-domain prototypical contrastive loss indirectly optimizes ProtoAN, enabling it to map ROI features to class-specific embedding spaces and ignore noise.

#### Remark 1

Notably, directly applying contrastive learning^35,36^ to cross-domain object detection faces two key challenges: (1) mining more same-class-positive/different-class-negative pairs with target domain information remains difficult even with high-confidence proposals; and (2) unlike classification, object detection proposal IoU is uncertain, and instances contain much noise. While our prototype-oriented contrastive learning has two properties: (1) it operates in a shared space dynamically learned by ProtoAN; and (2) it follows a clustering fashion, encouraged by prototype-oriented contrastive learning over intra- and inter-domain strong features.

In subsequent sections, we will elaborate on the design ideas and implementation details of the ProtoAN module and the Prototype-oriented Contrastive Learning Loss (PCL) in turn.

#### Prototype aligned network

Our ProtoAN design assumes an embedding space where each class’s ROI proposal projections cluster around a single prototype (or centroid). Here, inter-domain adaptation uses a prototype to represent each class distribution and aligns same-class prototypes in the embedding space learned from cross-domain proposals. ProtoAN can also extract unbiased features in more demanding settings (strong branch). Specifically, ProtoAN is a projection network with three sequential $3\times 3$ convolutional layers (no padding), each followed by a BN layer and a shortcut connection, ending with three fully connected layers. Its architecture is in Table 1.

**Table 1:** Architecture of ProtoAN.

Structure of network
Conv 2048 × 3 × 3, stride 2 → BatchNorm → ReLU
Conv 1024 × 1 × 1, stride 1 → BatchNorm → ReLU
Conv 1024 × 3 × 3, stride 2 → BatchNorm → ReLU
FC (1024 , 2048) → BatchNorm → ReLU
FC (2048 , 2048) → BatchNorm → ReLU
FC (2048 , 512)

Note that the design of ProtoAN’s architecture is tailored to the core demands of UDA-OD, effectively extracting compact, domain-invariant, and category-discriminative ROI features while balancing computational efficiency and adaptation performance. The specific rationale for this architecture is as follows:

- ResNet with feature pyramid network (FPN)^80^ serves as the core feature extraction backbone, which consists of five sequential stages (C1–C5) with progressively increasing channel dimensions (64, 256, 512, 1024, 2048 for C1 to C5, respectively). This FPN integration is critical for our PoCoMT framework’s UDA goal, as it enables effective multi-scale feature representation to mitigate domain shift across objects of different sizes, which is also compatible with ProtoAN’s input requirement of multi-scale ROI-aligned features.
- The three convolutional layers are designed to address feature dimensionality reduction, semantic aggregation, and domain gap mitigation, which are critical for UDA-OD. Empirically, fewer than 3 convolutional layers (e.g., 2 layers) fail to fully suppress domain-specific noise and aggregate semantic features, leading to suboptimal prototype quality. More than 3 layers (e.g., 4 layers) introduce excessive computational complexity without significant performance gains, violating the “lightweight plug-in” design goal of ProtoAN.
- The successive three FC layers are designed to map convolutional features to a prototype-aligned embedding space, balancing representation capacity and prototype discriminability: Empirically, 2 FC layers lack sufficient capacity to model cross-domain feature distributions, leading to prototype ambiguity; and 4 FC layers result in over-parameterization, causing the model to overfit to source domain features and degrade target domain adaptation. The 3-layer design strikes an optimal balance.
- The convolutional layers suppress domain-specific noise and spatial artifacts, while the FC layers map features to a domain-agnostic embedding space, aligning with the adversarial alignment insights and feature transferability exploration.

By exploiting ProtoAN, the ROI Features are projected into a 512-dimensional embedding space via an embedding layer, with hidden layer dimension 2048. ProtoAN inputs region features $\boldsymbol{r}_i \in \mathbb {R}^{H \times W \times C}$ from RoI operations (e.g., RoI Align^80^), denoted as $\mathcal {R} = \{\boldsymbol{r}_i\}_{i=1}^M$ (here, $M=300$ for both teacher and student). After ProtoAN mapping, weak/strong (ROI) features $\bar{x}_i$ and $\hat{x}_i$ become:

12 $$\begin{aligned} \bar{\boldsymbol{z}}_{i} = {\text {{ProtoAN}}}_\varTheta (\bar{{x}}_i),\quad \hat{\boldsymbol{z}}_{i} = {\text {{ProtoAN}}}_\varTheta (\hat{{x}}_i), \end{aligned}$$

where $\varTheta$ is ProtoAN’s parameters. Notably, these features are refined via weak-branch teacher proposals to retain key semantics.

#### Prototype computation in memory bank

In our PoCoMT, we leverage prototypes to retain domain-specific knowledge, which aids in distilling confident pseudo-labels from the source domain. These prototypes are dynamically updated using a memory bank that stores historical data.

##### Definition 1

(*Prototypical memory bank*) Suppose the prototypical memory bank $\mathcal {M}$ is a queue bundle that contains *K* queues with *D* size, in which *K* is the number of object categories of the training dataset, *D* is the storage length. We collectively push these queues to $\mathcal {M} \in \mathbb {R}^{K \times D}$. For the *k*th category ($k \in K$), the prototype in iteration *e*, denoted by $\mathcal {P}_k^{e}$, is computed as

13 $$\begin{aligned} \mathcal {P}_k^{e} = (1 - \theta ) \mathcal {P}_k^{e-1} + \theta \frac{1}{D} \sum _{i=1}^{D} \varTheta _{\text {{ProtoAN}}}(\mathcal {M}_{k,i}), \end{aligned}$$

where $\theta \in (0,1)$, $\mathcal {M}_{k,i}$ is the *i*-th element in the *k*-th row $\mathcal {M}_{k}$.

As shown in Eq. (13), $\mathcal {P}_k^{e}$ undergoes EMA-based updates, allowing it to capture the dynamics of adaptation. For updating the memory bank, we rely on the teacher’s predictions to select the Top-*D* high-confidence weak features for each category, which are then used to update $\mathcal {M}$.

Foreground prototype computation: As per Definition 1, the prototype update value for class *k* from the source or target domain can be derived as

14 $$\begin{aligned} \hat{q}_k^{s/t} =\frac{\sum _{i=1}^{D} 1\!\!1_{\left\{ \hat{c}_{i}^{s/t}=k\right\} } \hat{z}_i^{s/t}}{\sum _{i=1}^{D} 1\!\!1_{\left\{ \hat{c}_{i}^{s/t}=k\right\} }}. \end{aligned}$$

In the case of the target domain, which lacks ground-truth annotations, the pseudo labels obtained from the teacher network are employed to calculate the update value $\hat{q}_k^{t}$.

Let $\hat{\mu }_k^{s/t}$ represent the class prototype of the kth class in the source (s) or target (t) domain within the strong branch. Using the current update value $\hat{q}_k^{s/t}$, we update $\hat{\mu }_k^{s/t}$ through EMA as follows:

15 $$\begin{aligned} \hat{\mu }_k^{s/t} = \frac{\hat{\theta }\hat{\rho }_k^{s/t} \hat{\mu }_k^{s/t} +(1-\hat{\theta }) \hat{q}_k^{s/t}}{\hat{\rho }_k^{s/t}+1}, \end{aligned}$$

where $\hat{\theta }$ is a momentum term that can be empirically set as a constant, and the update count $\hat{\rho }_k^{s/t}$ is incremented after this operation. In a similar vein, the kth class prototype from the teacher branch can be computed as

16 $$\begin{aligned} \bar{\mu }_k = \frac{\bar{\theta }\bar{\rho }_k \bar{\mu }_k +(1-\bar{\theta }) \bar{q}_k}{\bar{\rho }_k+1}, \end{aligned}$$

where $\bar{\mu }_k =\frac{\sum _{i=1}^{D} 1\!\!1_{\left\{ \bar{c}_{i}=k\right\} } \bar{z}_i}{\sum _{i=1}^{D} 1\!\!1_{\left\{ \bar{c}_{i}=k\right\} }}$, $\bar{\theta }$ is a momentum term. Consequently, three prototypical memory banks $\hat{\mathcal {U}}^{{s}}$, $\hat{\mathcal {U}}^{{t}}$, and $\bar{\mathcal {U}}$ can be maintained for the source and target domains respectively:

17 $$\begin{aligned} \hat{\mathcal {U}}^{{s/t}}=\left[ \hat{\mathbf {\mu }}_{1}^{s/t}, \cdots , {\hat{\mu }_{K}^{s/t}}\right] , \bar{\mathcal {U}}=\left[ \bar{\mathbf {\mu }}_{1}, \cdots ,\bar{ \mathbf {\mu }}_{K}\right] . \end{aligned}$$

Background prototype computation: In contrast to earlier approaches, we regard the background as a separate $(K + 1)$th category, with its prototype identified through online weighted clustering. The computation of background prototype can be formulated as:

18 $$\begin{aligned} \begin{aligned} \hat{\mathcal {\mu }}_{K+1}^{s/t} = \frac{\sum _{\hat{\boldsymbol{x}}_i \in \hat{\boldsymbol{X}}} \left( \delta _{K+1}(\hat{c}_i){\hat{\boldsymbol{z}}_{i}}\right) }{\sum _{\hat{\boldsymbol{x}}_i \in \hat{\boldsymbol{X}}} \delta _{K+1}(\hat{c}_i)},\\ \bar{\mathcal {\mu }}_{K+1} = \frac{\sum _{\bar{\boldsymbol{x}}_i \in \bar{\boldsymbol{X}}} \left( \delta _{K+1}(\bar{c}_i){\bar{\boldsymbol{z}}_{i}}\right) }{\sum _{\bar{\boldsymbol{x}}_i \in \bar{\boldsymbol{X}}} \delta _{K+1}(\bar{c}_i)}, \end{aligned} \end{aligned}$$

where softmax function $\delta _{K+1}(\cdot )$ return the $(K+1)$-th element of the output vector.

Pseudo labels refinement: Given that the prototypes provide a classification bases, we implement the classification for the mapped features using similarity comparison in the two rounds below.

Round 1: Obtain prototypes-based pseudo label for $\bar{\boldsymbol{z}}_{i}$ by the nearest centroid measurement:

19 $$\begin{aligned} \bar{p}_i^t = \arg \min _{j} \mathcal {S}(D_{cos} (\bar{\boldsymbol{z}}_{i}, \bar{\mathcal {\mu }}_j)), j=1,...,K+1, \end{aligned}$$

where $\mathcal {S}(.)$ is the softmax operator, and $D_{cos}(,)$ computes the cosine distance of the two inputs.

Round 2: Obtain final pseudo label for $\bar{\boldsymbol{z}}_{i}$ by weighted clustering.

20 $$\begin{aligned} \begin{aligned} {\bar{\mu }}_{k}&= \frac{\sum _{\bar{\boldsymbol{x}}_i \in \bar{\boldsymbol{X}}} \left( 1\!\!1_{[\bar{p}_i^t=1]} \bar{\boldsymbol{z}}_{i}\right) }{\sum _{\bar{\boldsymbol{x}}_i \in \bar{\boldsymbol{X}}} 1\!\!1_{[\bar{p}_i^t=1]}}, \quad \bar{y}_k&= \arg \min _{k} \mathcal {S}(D_{cos} (\bar{\boldsymbol{z}}_i, {\bar{\mu }}_{k})), \quad k=1,...,K+1. \end{aligned} \end{aligned}$$

As mentioned above, our pseudo label refinement integrates the information from both prototypes (Eq. (19)) and the ProtoAN features (Eq. (20)).

#### Adaptation-aware prototypical contrastive losses

Standard contrastive learning uses InfoNCE loss^58^ for instance discrimination, roughly splitting into alignment (pulling positives) and uniformity (spreading negatives uniformly on unit hypersphere, preventing collapse). Though easing clustering collapse, negatives may cause class collision ^37^, harming clustering representations. Moreover, instance discrimination has a core flaw: learned representations lack data semantic structure, as instances from different samples are negatives regardless of semantics.

To tackle the aforementioned challenges, we implement adaptation-aware prototype-guided clustering using a memory bank that stores historical data. Within the shared embedding space projected by ProtoAN, we explore the clustering structure of target data via pseudo-labeling^26,81^ or self-supervised learning^31^. For this purpose, we first develop a prototypical contrastive loss (PCL). This loss promotes prototypical alignment across domains and prototypical uniformity, thereby maximizing the distance between clusters.

##### Definition 2

(*Prototype-oriented contrastive loss (PCL)*) Assume we obtain $K+1$ prototypes from the strongly augmented source/target domain after ROI Align operation, $\hat{\mathcal {U}}^{s/t}=\left\{ \hat{\boldsymbol{\mu }}_{1}^{s/t}, \hat{\boldsymbol{\mu }}_{2}^{s/t}, \ldots , \hat{\boldsymbol{\mu }}_{K+1}^{s/t}\right\}$, and another $K+1$ prototypes from the weakly augmented target domain, $\bar{\mathcal {U}}=\left\{ \bar{\boldsymbol{\mu }}_{1}, \bar{\boldsymbol{\mu }}_{2}, \ldots , \bar{\boldsymbol{\mu }}_{K+1}\right\}$ , our proposed PCL is defined as follows:

21 $$\begin{aligned} \begin{aligned} \mathcal {L}_{\textrm{PCL}}^{o_k\rightarrow \mu _k}&=\frac{1}{K} \sum _{k=1}^{K}-\log \frac{f_\tau (\boldsymbol{o}_{k},\mu _{k})}{f_\tau (\boldsymbol{o}_{k},\mu _{k})+\sum _{j=1, j \ne k}^{K+1} f_e(\boldsymbol{o}_{k},\mu _{j})} \\&\approx \underbrace{\frac{1}{K} \sum _{k=1}^{K}-\frac{dot(\boldsymbol{o}_{k}, \boldsymbol{\mu }_{k})}{\tau }}_{\text{ prototypical } \text{ alignment } }+\underbrace{\frac{1}{K} \sum _{k=1}^{K} \log \sum _{j=1, j \ne k}^{K+1} f_\tau (\boldsymbol{o}_{k},\mu _{j})}_{\text{ prototypical } \text{ uniformity } }, \end{aligned} \end{aligned}$$

where $f_{\tau }(x,y)={\exp \left( \frac{dot(x, y)}{\tau }\right) }$ with inner dot operator $dot(a,b) = a^Tb$ measures the exponential of cosine similarity, $o_k\in X^t \cup \hat{\mathcal {U}}^t\cup \bar{\mathcal {U}}$ and $\mu _k,\mu _j \in \hat{\mathcal {U}}^s \cup \hat{\mathcal {U}}^t$, and the temperature $\tau$ controls the concentration level of representations around a prototype.

Similarly, PCL (Eq.  (21)) roughly splits into prototypical alignment and uniformity. Prototypical alignment in PCL aligns a prototype with target features or another same-semantic prototype, stabilizing updates; uniformity pushes prototypes/features to uniform distribution over a unit hypersphere, maximizing inter-cluster distance and intra-cluster compactness. Intuitively, our prototype-focused PCL resembles traditional contrastive loss but avoids class collision (prototypes are inherent mutual negatives), suiting deep clustering better. Based on PCL definition, we introduce intra-domain and inter-domain PCL to realize feature-to-prototype and prototype-to-prototype contrastive learning, respectively.

Intra-domain prototypical contrastive loss: We concurrently perform intra-domain feature alignment for source and target domains in the strong branch, and intra-domain prototype alignment from the target’s weak to strong branch. In feature alignment, a feature forms a positive pair with its class prototype and negative pairs with other class prototypes. In prototype alignment, a target weak-branch prototype forms a positive pair with its matching class prototype and negative pairs with other class prototypes in the target strong branch. Specifically, the intra-domain feature alignment loss for feature $\hat{z}_{i(k)}^{s/t}$ (class *k*, prototype $\hat{\mu }^{s/t}_{k}$) is $\mathcal {L}_{\textrm{PCL}}^{\hat{z}_{i(k)}^{s/t}\rightarrow \hat{\mu }_k^{s/t}}$. Similarly, the intra-domain prototype alignment loss is $\mathcal {L}_{\textrm{PCL}}^{\bar{\mu }_{k}\rightarrow \hat{\mu }_k^t}$. Intra-domain PCL is the sum of these losses:

22 $$\begin{aligned} \begin{aligned} \mathcal {L}_{\mathrm {Intra-PCL}}&=\mathcal {L}_{\textrm{PCL}}^{\hat{z}_{i(k)}^{s}\rightarrow \hat{\mu }_k^s}+\mathcal {L}_{\textrm{PCL}}^{\hat{z}_{i(k)}^{t}\rightarrow \hat{\mu }_k^t}+\mathcal {L}_{\textrm{PCL}}^{\bar{\mu }_k\rightarrow \hat{\mu }_k^t},\\ \mathcal {L}_{\textrm{PCL}}^{\hat{z}_{i(k)}^{d}\rightarrow \hat{\mu }_k^d}&=\frac{-1}{K} \sum _{k=1}^{K}\log \frac{f_{\tau ^{d}_k}(\hat{\boldsymbol{z}}_{i(k)}^{d},\hat{{\mu }}_{k}^{d})}{f_{\tau ^{d}_k}(\hat{\boldsymbol{z}}_{i(k)}^{d},\hat{\mu }_{k}^{d})+\sum _{j \ne k}^{K+1} f_{\tau ^{d}_k}(\hat{\boldsymbol{z}}_{i(k)}^{d},\hat{\mu }_{j}^{d})}, \\ \mathcal {L}_{\textrm{PCL}}^{\bar{\mu }_k\rightarrow \hat{\mu }_k^{t}}&=\frac{-1}{K} \sum _{k=1}^{K}\log \frac{f_{\tau ^{t}_k}({\bar{\mu }}_{k},\hat{\mu }_{k}^{t})}{f_{\tau ^{t}_k}(\bar{\mu }_{k},\hat{\mu }_{k}^{t})+\sum _{j \ne k}^{K+1} f_{\tau ^{t}_k}(\bar{\mu }_{k}^{t},\hat{\mu }_{j}^{t})}, \end{aligned} \end{aligned}$$

where $$d \in \{ s,t \}$$.

Inter-domain prototypical contrastive loss: Our approach draws on the insight that same-category cross-domain samples should cluster in the latent space, but this only holds for the source domain (with labels). Target samples fail to align into clusters due to domain shift between target and source. This cross-domain cluster discrepancy for the same category can be reduced by aligning each class’s source and target prototypes; we also align target features with source prototypes. Concretely, a target feature $\hat{z}_{i}^t$ forms a positive pair with the same-class source prototype and a negative pair with other-class source prototypes. ProtoAN is trained to maximize similarity between source $\hat{\mu }_{k}^{s}$ and target $\hat{\mu }_{k}^{t}$ (positive pair), while other cluster prototypes from both domains form negative pairs pushed apart in the latent space. Inter-domain PCL is thus defined as:

23 $$\begin{aligned} \begin{aligned} \mathcal {L}_{\mathrm {Inter-PCL}}&=\mathcal {L}_{\textrm{PCL}}^{\hat{\mu }_k^t\rightarrow \hat{\mu }_k^s}+\mathcal {L}_{\textrm{PCL}}^{\hat{z}_{i(k)}^{t}\rightarrow \hat{\mu }_k^s}, \\ \mathcal {L}_{\textrm{PCL}}^{\hat{o}_k\rightarrow \hat{\mu }_k^s}&=\frac{1}{K} \sum _{k=1}^{K}-\log \frac{f_{\tau ^{s}_k}(\hat{o}_{k},\hat{\mu }_{k}^{s})}{f_{\tau ^{s}_k}(\hat{o}_{k},\hat{\mu }_{k}^{s})+\sum _{j=1, j \ne k}^{K} f_{\tau ^{s}_k}(\hat{o}_{k},\hat{\mu }_{j}^{s})}, \end{aligned} \end{aligned}$$

where $$\hat{o}_{k} \in \{ \hat{z}_{i(k)}^t, \hat{\mu }_k^t\}$$.

##### Remark 2

Current studies ^31,39^ generally focus on matching source and target features in a latent space, but our method differs: it represents each class’s domain features via a prototype and aligns features to their corresponding prototypes instead of other features. This strategy has advantages: first, prototype-oriented alignment is more robust to domain outliers, and notably, prototypes help correct incorrect pseudo-labels by downweighting false ones near the decision boundary (far from prototypes); second, using prototypes ensures all classes are represented in each training update, alleviating missing classes in domain-sampled minibatches (a problem in feature alignment), with prototypes treating classes equally regardless of frequency–valuable for object detection given class imbalance; finally, using deduced prototypes instead of features enables target adaptation without source data access.

**Algorithm 1:**
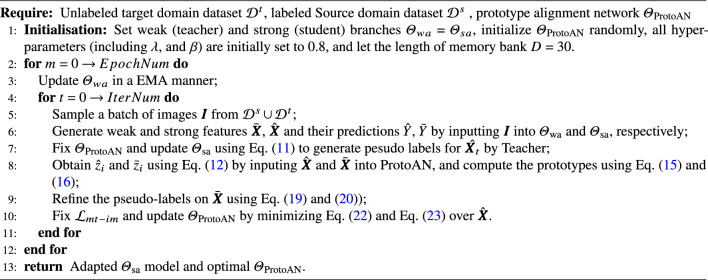
Training procedure of PoCoMT.

### Overall algorithm and inference

#### Training summary

This subsection outlines the PoCoMT training algorithm, which minimizes the total loss in  (1). The comprehensive loss $\mathcal {L}_\mathrm {mt-im}$ trains the student’s cross-domain feature encoder and detector; the teacher is updated only via EMA, and $\mathcal {L}_{pcl}$ updates the ProtoAN module. FasterRCNN is initialized with ImageNet pre-trained weights, starting with a burn-in phase using Eq. (1) for student training. Training details are in Algorithm  1 : each iteration has four steps, stopping at convergence. To address self-labeling inaccuracies, pseudo labels are assigned only to target instances with max scores>0.6; target instances are resampled per iteration to avoid pseudo-label overfitting. The PoCoMT training is robust to pseudo label noise, iteratively using labeled source and pseudo-labeled target instances to train the embedding function. This preserves source accuracy, reduces class/sample-level differences, and steadily improves target accuracy.

#### Target inference

During the inference stage, we can keep the target-specific Teacher model for predicting the target dataset as formulated in Eqs. (19) and (20). On the other hand, prototype-oriented contrastive learning module can further refine this inference result. Thereby, when the cross-domain mean-teacher model and ProtoAN have been obtained after training PoCoMT, it is reasonable to ensemble these two inference results from the Teacher and ProtoAN, respectively. In other words, we can leverage both domain prototypes rather than only the Teacher prototypes as formulated in Eqs. (19) and (20) for joint target inference, so as to further refine the target pseudo-labels. For EMA updates, the Teacher is a temporal ensemble of Student models with momentum, ensuring its superiority over the Student on the target domain ^82^, we therefore only retain Teacher and Source prototypes for pseudo-label inference.

Specifically, for target sample $x_i^t \in \mathcal {D}_t$, ProtoAN computes its clustering probability distribution over $K+1$ prototypes via softmax, based on cosine distances to $\left\{ \bar{\mu }_{k}\right\}$ and $\left\{ \hat{\mu }_{k}^{s}\right\}$ ($k=1,2,...,K+1$), respectively. The *k*-th component indicates the probability of belonging to class *k*, and the ensemble prediction for $x_i^t$ is formulated as:

24 $$\begin{aligned} {h}_{i k}^{t}= \omega \bar{ f}_k + (1-\omega ) \hat{f}_k^s, \quad k=1,...,K+1, \end{aligned}$$

where $\bar{f}_k=\frac{e^{-d\left( \bar{z}_i, \bar{\mu }_k\right) }}{\sum _{k} e^{-d\left( \bar{z}_i, \bar{\mu }_{k}\right) }}$, $\hat{f}_k^s=\frac{e^{-d\left( \hat{z}_i^t, \hat{\mu }_k^s\right) }}{\sum _{k} e^{-d\left( \hat{z}_i^t, \hat{\mu }_{k}^s\right) }}$ with cosine distance function $d(x_1, x_2) = (\frac{x^\textsf{T}_1 \cdot x_2}{\Vert x_1\Vert \Vert x_2\Vert } + 1) / 2,$

and $\omega$ denotes a balance factor that varies from 0 to 1. In this paper, we dynamically maintain this balance factor, which are adaptively updated by leveraging both prototypical classifier and Teacher classifier as:

25 $$\begin{aligned} \omega = d(\bar{f}_k, \hat{f}_k^s), \quad k=1,...,K=1. \end{aligned}$$

Finally, the label of the target sample $x_i^t$ can be deduced as follows:

26 $$\begin{aligned} {y}_{i}^{t}(k)=\operatorname {argmax}_{k}\left( h_{i k}^{t}\right) , \quad k=1,2,...K+1. \end{aligned}$$

By exploiting Eqs. (24)–(26), we can further refine the target pseudo-labels for boosting the quality of the target label set. In the nutshell, the complete inference pipeline are elaborated as follows:

- Prototype integration with RCNN classification. During inference, for each target sample $x_i^t \in \mathcal {D}^t$, we first feed it into the Teacher model and the trained ProtoAN module: The Teacher model (with EMA-stabilized weights) processes the weakly-augmented target sample to generate RPN proposals and their corresponding initial RCNN classification scores (category probabilities) and box regression offsets.The ProtoAN module extracts ROI features $\bar{z}_i$ (from weakly-augmented samples) and $\hat{z}_i^t$ (from strongly-augmented samples) for each RPN proposal, then computes the clustering probability distributions $\bar{f}_k$ (based on teacher prototypes $\left\{ \bar{\mu }_{k}\right\}$) and $\hat{f}_k^s$ (based on source prototypes $\left\{ \hat{\mu }_{k}^{s}\right\}$) via cosine distance and softmax, as formulated in Eq. (24). Crucially, the ensemble score $h_{i k}^{t}$ replaces the initial RCNN classification score of the Teacher model for each proposal. This replacement refines the category discriminability of each proposal, especially for ambiguous proposals affected by domain shift, as prototypes provide semantic grounding from both source and target domains.
- Box regression retention and post processing. The box regression branch of RCNN is not directly modified by the prototype module. We retain the box regression offsets predicted by the Teacher model for two reasons: The Teacher model, as a temporal ensemble of Student models with EMA momentum, has been optimized during training to predict accurate box offsets for target-domain objects (consistent with prior mean-teacher-based UDA-OD methods ^82^); our prototype-oriented contrastive learning primarily aims to mitigate intra/inter-domain semantic misalignment and refine category classification, while the box regression task relies more on spatial context and EMA-stabilized prediction, which is sufficiently robust in the Teacher model. After integrating the ensemble classification scores, we perform standard non-maximum suppression (NMS) processing: we apply a fixed IoU threshold to filter redundant proposals, using the refined ensemble scores $h_{i k}^{t}$ to rank proposals. Proposals with scores below a confidence threshold are discarded before NMS to reduce computational cost.
- Final label assignment. For the remaining proposals after NMS, we assign the final category label to each proposal using Eq. (26). The corresponding box coordinates are the ones predicted by the Teacher model’s RCNN regression branch, as they have been optimized to align with target-domain object layouts.

## Analysis

### Computational complexity

In this subsection, we will further conducted the computational complexity analysis for our proposed PoCoMT to clarify its efficiency advantages while maintaining superior performance.

Analysis of number of parameters: Our PoCoMT is built on the Faster R-CNN backbone with ResNet-50 as the feature extractor, consistent with most state-of-the-art UDA-OD methods for fair comparison, and introduces the prototype alignment network (ProtoAN) as a plug-in module. The number of model parameters includes: (1) baseline MT framework parameters$\approx$41.3M (ResNet-50:  23.5M; RPN and detection head:  17.8M); and (2) proposed ProtoAN module parameters$\approx$7.93M (accounting for shared weights across ROIs in UDA-OD tasks). Then the total parameters of PoCoMT$\approx$ 49.23M, representing a 19.20% increase compared to the baseline MT framework. This parameter growth aligns with the ProtoAN’s high-channel convolutional design, and is justified by the significant performance gains. Notably, it remains competitive with state-of-the-art methods that adopt complex prototype or contrastive modules (e.g., contrastive mean teacher^31^:  44.7M parameters, but with 2–3% lower mAP on UDA-OD benchmarks).

Analysis of FLOPs with ROI-wise computation. Our PoCoMT’s FLOPs are evaluated based on the standard input size, consistent with UDA-OD benchmark settings and the exact ProtoAN layer structure detailed in Table 1. The per-image complexity of the backbone (ResNet-50) and our ProtoAN (non-ROI-wise) is $\sim$15.2 billion FLOPs, resulting in $\sim$18 ms latency. Note that both the teacher and student branches in our model adopt the Faster-RCNN architecture with ProtoAN as the neck network. For each input image, the RPN generates approximately 300 RoIs, which are then fed into the RoI Align module to extract region-specific features. These ROI features are further processed by our proposed ProtoAN module and the subsequent RCNN head for classification and bounding box regression. The ROI-wise computation complexity primarily originates from three key components: RoI Align operation, ProtoAN module processing, and RCNN head computation for each ROI. We quantify the FLOPs and latency contribution of each component, along with the multiplicative effect of 300 RoIs per image as follows (all measurements are conducted on an NVIDIA A100 GPU with batch size 1) .

1. RoI Align (300 RoIs): $\sim$120.42 million FLOPs, and $\sim$6 ms latency;
2. ProtoAN (300 RoIs): $\sim$110.23 billion FLOPs, and $\sim$45 ms latency;
3. RCNN Head (300 RoIs): $\sim$322.2 million FLOPs, and $\sim$1.5 ms latency.

Summing these three ROI-wise components and adding the complexity of the backbone (ResNet-50) and ProtoAN (non-ROI-wise), we present the complete per-image FLOPs and latency breakdown: Total per-image FLOPs is $\sim$125.87 billion FLOPs (the ROI-wise components contribute $\sim$110.67 billion FLOPs, accounting for  87.9% of the total FLOPs); and Total per-image latency is  70.5 ms (the ROI-wise components contribute  52.5 ms, accounting for  74.5% of the total latency).

Analysis of training time. Training time evaluations were conducted on a single NVIDIA A100 GPU with identical hyperparameters (batch size=4, learning rate=0.001, and total epochs=120) across all compared methods, using the Cityscapes$\rightarrow$Foggy Cityscapes benchmark (a typical UDA-OD scenario with large domain shift). The results are as follows:

1. Baseline MT framework:  28.5 h of total training time (average  14.25 min per epoch);
2. Our PoCoMT:  35.2 h of total training time (average  17.6 min per epoch), a 23.51% increase compared to the baseline. The growth in training time is consistent with the parameter count and FLOPs of ProtoAN, resulting from additional computations in convolutional/FC layers and associated prototype alignment. To optimize efficiency, ProtoAN shares layer parameters across all ROI features, avoiding per-ROI redundant computations and mitigating excessive training time overhead;
3. Comparison with state-of-the-art methods: our framework exhibits higher efficiency than most state-of-the-art methods with comparable or better performance (e.g., contrastive mean teacher^31^:  36.8 h, a 29.1% increase compared to the baseline).

The computational complexity analysis confirms that our PoCoMT achieves a favorable trade-off between computational cost and adaptation performance. Compared to the baseline MT framework, our method introduces increases in parameters, FLOPs, and training time while achieving 4.2 6.8% mAP gains on multiple UDA-OD benchmarks (Cityscapes$\rightarrow$Foggy Cityscapes, SIM10k$\rightarrow$Cityscapes, KITTI$\rightarrow$Cityscapes). This trade-off is superior to that of competing methods: our PoCoMT delivers higher mAP than methods with lower computational overhead and maintains lower FLOPs/parameters than methods with comparable performance (e.g., Contrastive Mean Teacher^31^). The structured design of ProtoAN ensures that the additional computational cost directly translates to enhanced prototype alignment and cross-domain feature discriminability, justifying the overhead for practical scenarios requiring high-precision object detection.

### Clustering concentration estimation

Intuitively, embedded features around each prototype may show varying concentration levels, estimated by $\tau _k^{s/t}$ ($k=1,2,...,K$) for class *k*. Smaller $\tau _k^{s/t}$ indicates lower dispersion (higher concentration) of samples around the prototype. $\tau _k^d$ is calculated using strongly augmented features $\hat{z}_{ik}^{d}$ ($d \in \{s,t\}$) in the same cluster as prototype $\hat{\mu }_k^d$:

27 $$\begin{aligned} \tau _k^d = \frac{\sum _{i=1}^{D}1\!\!1_{\{\hat{c}_i^d=k\}}\Vert \hat{z}_{ik}^d - \mu _k^d\Vert _2^2}{n_k^d \log (n_k^d + \delta _k^d)}, \quad k=1,2,...,K, \, d\in \{s,t\}, \end{aligned}$$

where $n_k^d = \sum _{i=1}^{D}1\!\!1_{\{\hat{c}_i^d=k\}}$, and smoothing parameter $\delta _k^d = \frac{1}{\sqrt{2\pi }}e^{-|n_k^d|}$ prevents small clusters from inflating $\tau _k^d$.

In $\mathcal {L}_{\text {Intra-PCL}}$, $\tau _k^d$ scales the similarity between ROI feature $\hat{z}_{ik}^d$ and its prototype $\mu _k^d$: larger $\tau _k^d$ (looser clusters) means lower similarity. Minimizing $\mathcal {L}_{\text {Intra-PCL}}$ aligns ROI features closer to their prototypes, while minimizing $\mathcal {L}_{\text {Inter-PCL}}$ improves cross-domain ROI feature distribution alignment. Jointly minimizing these losses via $\tau _k^d$ tuning balances clustering discriminativity and transferability.

### Generalization bound

Building upon the existing theory^83^, we formally analyze PoCoMT’s generalization performance. Given its reliance on both labeled source data and pseudo-labeled target data, the learning error is formulated as a weighted combination:

28 $$\begin{aligned} \epsilon _{\hat{\eta }}(h) = \hat{\eta } \epsilon _{t}(h, \hat{g}^{t}) + (1-\hat{\eta }) \epsilon _{s}(h, g^{s}), \end{aligned}$$

where $\hat{\eta }$ balances contributions from the target and source domains. Here, $\epsilon _{t}(h, \hat{g}^{t})$ and $\epsilon _{s}(h, g^{s})$ denote expected errors under the pseudo-label and ground-truth label functions, respectively. The oracle error $\epsilon _{t}(h, g^{t})$ measures performance against the target domain’s true labels. The efficacy of domain adaptation hinges on minimizing the gap between $\epsilon _{\hat{\eta }}(h)$ and $\epsilon _{t}(h, g^{t})$. The following lemma bounds this generalization gap:

#### Theorem 1

(Generalization bound) For any $h \in \mathcal {H}$,

29 $$\begin{aligned} |\epsilon _{\hat{\eta }}(h) - \epsilon _{t}(h, g^{t})| \le (1-\hat{\eta })\left( \frac{1}{2} d_{\mathcal {H} \Delta \mathcal {H}}(\mathcal {D}^{s}, \mathcal {D}^{t}) + \varsigma \right) + \hat{\eta } \hat{\rho }, \end{aligned}$$

where $d_{\mathcal {H} \Delta \mathcal {H}}(\mathcal {D}^{s}, \mathcal {D}^{t})$ measures domain discrepancy, $\hat{\rho }$ denotes the proportion of mislabeled target samples, and $\varsigma = \epsilon _{s}(h^{*}, g^{s}) + \epsilon _{t}(h^{*}, g^{t})$ with $h^{*} = \arg \min _{h \in \mathcal {H}} \left( \epsilon _{s}(h, g^{s}) + \epsilon _{t}(h, g^{t})\right)$.

The bound decomposes into three terms: domain divergence $d_{\mathcal {H} \Delta \mathcal {H}}$, optimal hypothesis error $\varsigma$, and pseudo-label noise $\hat{\rho }$. PoCoMT addresses these via: (1) class-level divergence $\mathcal {L}_{pcl}$ and adversarial consistency $\mathcal {L}_{adv}$ to minimize $d_{\mathcal {H} \Delta \mathcal {H}}$; (2) information maximization $\mathcal {L}_{IM}$ to refine pseudo-labels, reducing $\hat{\rho }$ iteratively; (3) assuming $\varsigma$ is negligible, a common approximation in domain adaptation. Through these mechanisms, PoCoMT effectively tightens the generalization bound in Eq. (29).

## Experiments

### Dataset

The proposed approach was evaluated on 7 cross-domain tasks across 4 scenarios using the following 8 datasets:

- Cityscapes^84^: is a street scene dataset comprising 2975 training images and 500 validation images, gathered from 50 distinct cities. For the object detection task, we select 8 object categories, with bounding boxes derived from segmentation masks.
- Foggy-Cityscapes^85^: is a synthetic dataset generated by introducing fog into the original Cityscapes images. Three fog intensity levels (0.02, 0.01, 0.005) are simulated, each corresponding to varying visibility ranges. In our experiments, we utilize both the most challenging 0.02 fog split and all available splits.
- Pascal VOC^86^: is a dataset featuring 20 categories of common objects in real-world scenes. Following^5^’s split, source domain uses 16,551 images from PASCAL VOC 2007/2012 (training/validation).
- Clipart^49^: consists of clip art images and shares the same category set as Pascal VOC, but differs in image stylistic characteristics. Both the training and validation splits of Clipart contain 500 images each.
- Watercolor^49^: 1K training/1K test images (6 categories). For Pascal$\rightarrow$Watercolor adaptation, source model uses only 6 shared categories per^87^.
- KITTI^88^: is an additional street scene dataset, though its images are captured with cameras in cities that differ from those used for Cityscapes data collection. For domain adaptation experiments, we employ the training split of KITTI (7,481 images) and only focus on the “car” category– the sole category shared between KITTI and Cityscapes.
- Sim10K^89^: 10K synthetic car images (GTA5) with 58,701 bounding boxes. For Sim10k$\rightarrow$Cityscapes adaptation, car category performance is reported per standard target domain settings.
- BDD100k^90^: consists of 100k images which are split into training, validation, and testing sets. There are 70k training images and 10k validation images with available annotations. This dataset includes different interesting attributes; there are 6 types of weather, 6 different scenes, 3 categories for the time of day and 10 object categories with bounding box annotation.

### Implementation details.

For fairness, we follow prior experimental setups^25,87^ with Faster RCNN as the base detector and ResNet^91^ with FPN (pre-trained on ImageNet^92^) as the backbone. Following the implementation of Faster RCNN with ROI-alignment^80^, all input images are resized to 800 pixels on the shorter side. Our framework uses EMA momentum values $\rho = 0.9996$ (teacher network) and $\hat{\theta }(\bar{\theta }) = 0.99$ (prototype updates), with the teacher generating 300 proposals and a 0.9 high-confidence threshold. For the initialization stage of training the framework, we train the PoCoMT using the source labels for 10k iterations. Then we copy the weights to both Teacher and Student models in the beginning of mutual learning and train our PoCoMT for 50k iterations. The student model is trained via SGD (learning rate 0.001). Testing reports teacher network mAP on the target domain (IoU = 0.5). Experiments ran on four NVIDIA A100 GPUs using PyTorch and Detectron2^93^.

In the case of AT^19^, it is noted that certain objects are fully eliminated from the student’s perspective because of the strong augmentation introduced by Cutout^94,95^. Under these circumstances, compelling their features to match those in the teacher’s view loses its significance. Such objects should be excluded based on an empirical standard^31^: within each object’s bounding box, we tally pixels where the RGB value discrepancy between the teacher’s and student’s views goes beyond 40. When the proportion of these pixels is over 50%, the object is deemed removed by Cutout^94^ and left out of our prototype-based contrastive learning.

### Experimental settings and evaluation

Following prior works^19,20,31^, eight datasets form seven transfer tasks across four scenarios: (1) *Adverse weather adaptation*: Cityscapes $\rightarrow$ FoggyCityscapes; (2) *City scene adaptation*: cross-camera (KITTI $\rightarrow$ Cityscapes) and synthetic-to-real (Sim10k $\rightarrow$ Cityscapes); (3) *Realistic-to-artistic adaptation*: watercolor (Pascal $\rightarrow$ Watercolor) and clipart (Pascal $\rightarrow$ Clipart);  (4) *Cross time adaptation*: BDD100k (daytime $\rightarrow$ dawn) and BDD100k (nighttime $\rightarrow$ dawn).

Unsupervised domain adaptation uses source/target training splits; target validation splits assess performance. Comparisons use mAP (per Pascal VOC standards). We use the current State-Of-The-Art, Adaptive Teacher (AT)^19^ and Probabilistic Teacher (PT)^20^, as baselines, integrating our ProtoAN to create PT^20^+ProtoAN and AT^19^+ProtoAN. PoCoMT’s effectiveness is evaluated against three groups: (1) source (Faster R-CNN trained on the labeled source data) and Oracle (Faster R-CNN trained on target data with the ground-truth annotations, which are unavailable in the training stage) models, which specify the lower and upper bounds of transfer performance, respectively; (2) representative UDA-OD methods (results from original papers); and (3) our method and variants: PoCoMT, PT^20^+ProtoAN, AT^19^+ProtoAN.

For a fair comparison, we first reproduce two baselines AT^19^ and PT^20^ using their released codes with and without ProtoAN, respectively. We also re-run the public codes of UMT^13^, SWDA^5^, SCDA^4^, AT^19^+CMT^31^, SimROD^96^, PDA^52^, TIA^97^, and PT^20^+CMT^31^, and we implement PoCoMT by ourselves. For the rest methods included in the comparison, the originally reported results are collected from their corresponding papers if available. Besides, we run Faster R-CNN (FRCNN^2^) with the provided source codes via Detectron2^93^.

**Table 2:** Domain adaptation from normal weather (Cityscapes) to adverse weather (Foggy Cityscapes). Best in bold.

Method	Split	Person	Rider	Car	Truck	Bus	Train	Motor	Bike	mAP
Source	0.02	22.4	26.6	28.5	9.0	16.0	4.3	15.2	25.3	18.4
Oracle	0.02	39.5	47.3	59.1	33.1	47.3	42.9	38.1	40.8	43.5
DM^54^	0.02	30.8	40.5	40.5	27.2	38.4	34.5	28.4	32.3	34.6
HTCN^8^	0.02	33.2	47.5	47.9	31.6	47.4	40.9	32.3	37.1	39.8
MeGA-CDA^48^	0.02	37.7	49.0	52.4	25.4	49.2	**46.9**	34.5	39.0	41.8
TIA^97^	0.02	34.8	46.3	49.7	31.1	52.1	48.6	37.7	38.1	42.3
SIGMA^98^	0.02	**46.9**	48.4	63.7	27.1	50.7	35.9	34.7	41.4	43.5
DAF^3^	0.02	25.0	31.0	40.5	22.1	35.3	20.2	20.0	27.1	27.6
SWDA^5^	0.02	29.9	42.3	43.5	24.5	36.2	32.6	30.0	34.8	34.3
MAF^6^	0.02	28.2	39.5	43.9	23.8	39.9	33.3	29.2	33.9	34.0
SCL^99^	0.02	41.8	36.2	44.8	33.6	31.6	44.0	40.7	30.4	37.9
C2F^79^	0.02	35.1	42.1	49.1	30.0	45.2	26.9	26.8	36.0	36.4
SCDA^4^	0.02	33.8	42.1	52.1	26.8	42.5	26.5	29.2	34.5	35.9
MTOR^18^	0.02	30.6	41.4	44.0	21.9	43.4	40.2	31.7	33.2	35.1
UMT^13^	0.02	33.0	46.7	48.6	34.1	56.5	46.8	30.4	37.3	41.7
PT^20^	0.02	40.2	48.8	59.7	30.7	51.8	30.6	35.4	44.5	42.7
AT^19^	0.02	45.3	55.7	63.6	36.8	64.9	34.9	42.1	51.3	49.3
AT^19^ + CMT^31^	0.02	45.9	55.7	63.7	39.6	66.0	38.8	41.4	51.2	50.3
PT^20^ + ProtoAN (Ours)	0.02	43.3	53.1	62.1	33.9	56.7	37.4	42.5	47.6	47.1
AT^19^ + ProtoAN (Ours)	0.02	45.9	56.2	65.3	41.4	66.5	37.7	43.1	53.1	51.2
PoCoMT (Ours)	0.02	47.5	**56.9**	**65.8**	**41.7**	**68.2**	39.3	**44.6**	**53.9**	**52.2**
Source	All	27.9	33.4	40.4	12.1	23.2	10.1	20.7	30.9	24.8
Oracle	All	41.2	49.1	61.6	32.6	56.6	49.0	37.9	42.4	46.3
PDA^52^	All	36.0	45.5	54.4	24.3	44.1	25.8	29.1	35.9	36.9
ICR-CCR^7^	All	32.9	43.8	49.2	27.2	36.4	36.4	30.3	34.6	37.4
PT^20^	All	43.2	52.4	63.4	33.4	56.6	37.8	41.3	48.7	47.1
AT^19^	All	46.3	55.9	64.3	38.5	61.1	39.3	40.8	52.3	49.8
AT^19^ + CMT^31^	All	47.0	55.7	64.5	39.4	63.2	51.9	40.3	53.1	51.9
PT^20^ + ProtoAN (Ours)	All	46.5	55.6	65.1	36.7	60.6	42.5	**41.9**	51.4	50.0
AT^19^ + ProtoAN (Ours)	All	50.0	58.3	64.8	41.3	65.8	52.1	41.0	55.6	53.6
PoCoMT (ours)	All	**51.4**	**59.8**	**65.4**	**44.9**	**66.1**	**52.7**	41.5	**57.2**	**54.9**

**Table 3:** Results on Sim10k $\rightarrow$ Cityscapes and KITTI $\rightarrow$ Cityscapes. Best in bold.

	Sim10K $\rightarrow$ City		KITTI $\rightarrow$ City	
Method	AP on car	Gains w.r.t source	AP on car	Gains w.r.t source
Source	35.5	–	40.3	–
Oracle	66.4	–	66.4	–
DAF^3^	38.9	+3.4	38.5	-1.8
SWDA^5^	40.1	+4.6	37.9	-2.4
SCDA^4^	43.0	+7.5	42.5	+2.2
MAF^6^	41.1	+5.6	41.0	+0.7
UMT^13^	43.1	+7.6	–	–
MeGA^48^	44.8	+9.3	43.0	+2.7
TIA^97^	–	–	44.0	+3.7
SIGMA^98^	–	–	45.8	+5.5
SimROD^96^	52.1	+16.6	47.5	+7.2
C2F^79^	–	–	52.1	+11.8
PT^20^	55.1	+19.6	60.2	+19.9
PT^20^ + CMT^31^	56.3	+20.8	64.3	+24.0
PT^20^ + ProtoAN (ours)	57.4	+21.9	62.7	+24.0
PoCoMT (ours)	**59.5**	**+24.0**	**64.7**	**+24.4**

**Table 4:** Domain adaptation from realistic images (Pascal VOC) to artistic images (Clipart1k). Best in bold.

		Pascal $\rightarrow$ clipart																			
Method	Aero	Bicycle	Bird	Boat	Bottle	Bus	Car	Cat	Chair	Cow	Table	Dog	Horse	Bike	Prsn	Plnt	Sheep	Sofa	Train	Tv	mAP
Source	23.0	39.6	20.1	23.6	25.7	42.6	25.2	0.9	41.2	25.6	23.7	11.2	28.2	49.5	45.2	46.9	9.1	22.3	38.9	31.5	28.8
Oracle	33.3	47.6	43.1	38.0	24.5	82.0	57.4	22.9	48.4	49.2	37.9	46.4	41.1	54.0	73.7	39.5	36.7	19.1	53.2	52.9	45.0
SWDA^5^	26.2	48.5	32.6	33.7	38.5	54.3	37.1	**18.6**	34.8	58.3	17.0	12.5	33.8	65.5	61.6	52.0	9.3	24.9	54.1	49.1	38.1
SAPNet^100^	27.4	**70.8**	32.0	27.9	42.4	63.5	47.5	14.3	48.2	46.1	31.8	17.9	43.8	68.0	68.1	49.0	18.7	20.4	55.8	51.3	42.2
PD^101^	41.5	52.7	34.5	28.1	43.7	58.5	41.8	15.3	40.1	54.4	26.7	28.5	37.7	75.4	63.7	48.7	16.5	30.8	54.5	48.7	42.1
ICR-CCR^7^	28.7	55.3	31.8	26.0	40.1	63.6	36.6	9.4	38.7	49.3	17.6	14.1	33.3	74.3	61.3	46.3	22.3	24.3	49.1	44.3	38.3
HTCN^8^	33.6	58.9	34.0	23.4	45.6	57.0	39.8	12.0	39.7	51.3	20.1	20.1	39.1	72.8	61.3	43.1	19.3	30.1	50.2	51.8	40.3
DM^54^	25.8	63.2	24.5	42.4	47.9	43.1	37.5	9.1	47.0	46.7	26.8	24.9	48.1	78.7	63.0	45.0	21.3	36.1	52.3	**53.4**	41.8
UMT^13^	39.6	59.1	32.4	35.0	45.1	61.9	48.4	7.5	46.0	**67.6**	21.4	29.5	48.2	75.9	70.5	56.7	25.9	28.9	39.4	43.6	44.1
TIA^97^	42.2	66.0	36.9	37.3	43.7	**71.8**	49.7	18.2	44.9	58.9	18.2	29.1	40.7	**87.8**	67.4	49.7	**27.4**	27.8	**57.1**	50.6	46.3
AT^19^	33.1	66.1	35.3	44.9	57.5	44.9	51.0	5.8	59.5	54.9	34.6	23.5	64.3	84.0	75.4	51.5	17.1	30.3	43.3	37.2	45.7
AT^19^ + CMT^31^	39.8	56.3	38.7	39.7	60.4	35.0	56.0	7.1	60.1	60.4	35.8	28.1	67.8	84.5	80.1	55.5	20.3	32.8	42.3	38.2	47.0
AT^19^ + ProtoAN(ours)	41.3	57.7	39.5	45.7	60.8	57.4	59.2	13.7	61.5	63.5	37.6	31.3	69.2	84.9	81.0	56.2	25.4	35.1	47.5	48.3	50.8
PoCoMT(ours)	**42.8**	58.3	**41.2**	**46.2**	**61.5**	64.9	**61.4**	14.3	**61.8**	65.0	**39.1**	**31.6**	**71.7**	85.2	**81.8**	**57.4**	27.3	**37.0**	52.6	52.5	**52.7**

**Table 5:** The results of cross-domain object detection on PASCAL VOC $\rightarrow$ Watercolor2k adaptation. Best in bold.

Method	Bicycle	Bird	Car	Cat	Dog	Person	mAP
Source	84.2	44.5	53.0	24.9	18.8	56.3	46.9
Oracle	51.8	49.7	42.5	38.7	52.1	68.6	50.6
SCL^99^	82.2	55.1	51.8	39.6	38.4	64.0	55.2
SAPNet^100^	81.1	51.1	53.6	34.3	39.8	71.3	55.2
PD^101^	95.8	54.3	48.3	42.4	35.1	65.8	56.9
SWDA^5^	82.3	55.9	46.5	32.7	35.5	66.7	53.3
UMT^13^	88.2	55.3	51.7	**39.8**	**43.6**	69.9	58.1
AT^19^	93.6	56.1	58.9	37.3	39.6	73.8	59.9
AT^19^+CMT^31^	93.9	56.8	58.2	38.5	41.4	74.7	60.6
AT^19^+ProtoAN(ours)	94.2	58.3	60.4	38.5	41.8	73.2	61.1
PoCoMT (ours)	**94.8**	**59**	**61.7**	38.7	43.5	74.6	**62.1**

#### Adverse weather adaptation

Object detectors in real applications often face weather conditions differing from training. For example, rain, snow, or fog degrades camera-captured image quality, challenging detector performance. Thus, domain adaptation bridges the shift from normal to adverse weather. Here, PoCoMT is tested on the standard Cityscapes $\rightarrow$ Foggy Cityscapes benchmark, requiring adaptation from normal weather to low-visibility foggy scenes. Experimental results in Table 2 are provided for training and testing on both the most fog-dense images (“0.02” subset) and all synthetic images (“All” subset) within Foggy Cityscapes.

As mentioned-above, cross-domain mean-teacher self-training approaches, such as AT^19^, PT^20^, and CMT^31^, lead the field in UDA-OD. These approaches not only surpass the capabilities of earlier methods that do not employ the mean-teacher framework but also outperform the so-called “Oracle” models. Their exceptional performance can be attributed to their unique capacity to leverage datasets from both source and target domains, facilitating the transfer of knowledge between different domains.

Our AT^19^+ProtoAN and PT^20^+ProtoAN yield additional performance improvements over AT^19^ and PT^20^, respectively, even outperforming AT^19^+CMT^31^ in several settings. On both the “0.02” and “All” subsets, consistent enhancements are seen for our PoCoMT and obtain the best mAP performance, achieving current state-of-the-art results. To be concrete, our AT^19^+ProtoAN (respectively PT^20^+ProtoAN) boosts the previous best from AT^19^ alone (PT^20^ alone) by +1.9% mAP (+4.4% mAP) on the “0.02” subset and +3.8% mAP (+2.9% mAP) on the “All” subset, respectively. A larger improvement from PoCoMT is observed on the “All” subset compared to “0.02”, highlighting its strong capacity to learn robust features from increased unlabeled data. In real-world settings, unlabeled data is often abundant, but labeling costs are high. Ideally, domain adaptation methods should continuously enhance target-domain performance as unlabeled training data expands, and our PoCoMT is well-suited to this task.

#### City scene adaptation

Cross-camera scene adaptation: Sensors used in real settings, such as cameras, often have highly varied setups (e.g., intrinsic parameters, resolution levels), and these variations can harm the performance of deployed object detectors. Furthermore, Cityscapes, gathered from various urban areas distinct from KITTI’s sources, features more diverse street scenes, making the task more demanding. To examine its efficacy in cross-camera adaptation, PoCoMT is evaluated using the KITTI $\rightarrow$ Cityscapes domain adaptation benchmark. Following earlier research practices^19,20^, object detectors are trained and tested solely for the shared “Car” category present in both KITTI and Cityscapes. Comparisons of outcomes are provided in Table 3. The cross-domain mean-teacher self-training approach PT^20^+CMT^31^ outperforms all prior methods by a significant margin. When integrated with our proposed ProtoAN framework, PT ^20^ gains an extra 2.5% AP in performance. Our proposed method PoCoMTimproves upon the best method PT+CMT^31^ by +0.4 mAP. Notably, our method also outperforms the very recent work SimROD, which takes YOLO^1^ as the base detector and relies on a large-scale teacher model.

Synthetic to real scene adaptation: Synthetic imagery provides another way to ease issues related to data gathering and labeling. That said, a distribution mismatch exists between synthetic and real-world data. For adapting synthetic scenes to real ones, the full Sim10k dataset serves as source data, with Cityscapes’ training set acting as target data. Given that only the *car* category has annotations in both domains, we present the AP of car from Cityscapes’ test set.

As shown in Table 3 , Similar trends can be observed as cross-camera adaptation setting for our methods PoCoMTand PT^20^+ProtoAN. Concretely, our PT^20^+ProtoAN attains an extra 2.3% AP gains over PT^20^ alone in performance. Our method PoCoMT consistently surpasses the second best method PT^20^+CMT^31^ by a substantial margin of +3.3 AP.

#### Realistic to artistic adaptation

In this setting, we aim to assess how effectively our model handles significant domain discrepancies. Here, we examine a domain adaptation task involving shifts in image style, specifically, from realistic images to artistic ones. Pascal VOC serves as the source dataset, with Clipart1k or Watercolor2k as target datasets. Our method is compared against multiple leading approaches, and we document the performance difference between the oracle model (fully supervised) and each competing method.

Results for real-to-artistic adaptation on Clipart1k are shown in Table 4, and those on Watercolor2k appear in Table 5. The mean-teacher self-training methods are leading unsupervised domain adaptation for object detection. They not only outperform previous non mean-teacher methods, but also surpass the “Oracle” models. The reason is that they can efffectively leverage the images from the both source and target domains, and transfer cross-domain knowledge, which is consistent with preceding works^19,20,31^. Our model PoCoMT achieves state-of-the-art results at 52.7% mAP on Clipart and 62.1% on Watercolor, outperforming the recent competitor AT^19^+CMT^31^ by 5.7% and 1.5%, repsectively, and other methods by a considerable margin. The AT^19^ + ProtoAN combination enhances AT by 5.1% mAP on Clipart and 1.2% mAP on Watercolor, and surpasses the previous top performer AT^97^+CMT^31^ by 3.8% mAP and 0.5% mAP, respectively. Notably, UMT, which uses Mean Teacher, already saw notable gains through training with style-augmented images. However, due to inherent problems with the quality of pseudo labels generated by Mean Teacher on the target domain, their model may still struggle with large domain shifts between real and artistic images during pseudo label creation. In contrast, our model PoCoMT reduces this domain gap, leading to substantial performance improvements by 8.6% mAP on Clipart and by 4.0% on Watercolor. Note that Oracle results are slightly worse than Source results for some classes (e.g., bicycle) in Table 5, consistent with that reported in the referenced works^19,20,31^. The main reason is that although the Oracle model is trained on target data with ground-truth annotations, the insufficient sample size of specific classes in the target dataset leads to underfitting. In contrast, the Source model is trained on a large number of labeled source samples and has learned more generalized feature representations for these classes, thus achieving slightly better performance on these specific classes than the Oracle model.

**Table 6:** Detailed results for the setting of cross time adaptation on BDD100K. mAP (%) for all the classes and detailed AP (%) of each individual category on BDD100K *dawn/dusk* are reported. Best in bold.

Source	Method	Bike	Bus	Car	Motor	Person	Rider	Light	Sign	Truck	mAP
Day	SWDA^5^	34.9	51.2	52.7	15.1	32.8	23.6	21.6	35.6	47.1	31.4
	SCL^99^	29.1	51.3	52.8	17.2	32.0	19.1	21.8	36.3	47.2	30.7
	ICR-CCR^7^	32.8	51.4	53.0	15.4	32.5	22.3	21.2	35.4	**47.9**	31.2
	UMT^13^	39.7	52.3	56.1	14.2	35.7	23.7	31.5	42.2	42.4	33.8
	AT^19^	38.2	52.4	56.8	17.1	36.2	23.9	31.8	42.5	46.4	38.4
	AT^19^+CMT^31^	39.4	52.6	57.3	18.6	36.2	24.0	33.7	42.5	47.1	39.0
	**PoCoMT (Ours)**	**41.8**	**54.7**	**58.8**	**20.6**	**37.8**	**24.7**	**35.0**	**43.4**	46.7	**40.4**
Night	SWDA^5^	31.4	38.2	51.0	9.9	29.5	22.2	18.7	32.5	35.7	26.9
	SCL^99^	25.3	31.7	49.3	8.9	25.8	21.2	15.0	28.6	26.2	23.2
	ICR-CCR^7^	32.3	45.1	51.6	7.2	29.2	24.9	19.9	33.0	41.1	28.4
	UMT^13^	37.9	18.4	50.4	8.8	24.7	11.6	15.1	30.1	19.4	21.6
	AT^19^	42.5	46.9	54.6	14.3	32.4	23.7	24.6	31.5	35.7	34.0
	AT^19^+CMT^31^	42.9	48.6	56.0	15.7	33.4	25.2	30.2	34.5	38.7	36.1
	**PoCoMT (Ours)**	**44.8**	**52.8**	**59.6**	**17.1**	**37.5**	**26.8**	**35.2**	**36.8**	**43.5**	**39.3**
Oracle	FRCNN^2^	27.2	39.6	51.9	12.7	29.0	15.2	20.0	33.1	37.5	26.6

#### Cross time adaptation

In real-world applications, a detector is often deployed at different time, where changes in illumination and scene can be extremely large. To evaluate the performance of our method against such a factor on BDD100k^90^, we follow the setting in^102^ to adapt knowledge learned in the daytime and nighttime to corner cases, i.e. at dawn or dusk. Concretely, BDD100K^90^ is divided into three subsets by time, including *daytime* (Day), *nighttime* (Night), *dawn/dusk*. 36,728 images in the Day and 27,971 images at Night constitute two source domains. Images collected by excluding the ones in Day and Night are relatively few, where 5,027 unlabeled images are used for training and 778 validation images for evaluation at *dawn/dusk* as the target domain. The mean average precision (mAP) over 9 categories is reported for comparison (by following^102^, the result on the category “train” is not reported).

The mAP performance of our method compared against the other approaches on cross time adaptation is summarized in Table 6 . As in Table 6, previous UDA-OD methods fail to improve the detection performance when using images from the Day and Night, respectively, due to the large domain discrepancy between source and target domains. Our PoCoMT improves the detection performance for almost all the categories and achieve the best result when different data source is adopted, respectively. It can also be seen that the performance of our PoCoMT method is much better than the Oracle Target-Only case, which is owing to insufficient training images in the target domain.

Specifically, preliminary results can be observed from the Table 6 :

- On Day$\rightarrow$Dawn/Dusk, our framework achieves 40.4% mAP, outperforming the second-best method (AT^19^+CMT^31^, 39.0% mAP) by 1.4% mAP. This gain stems from our prototype-guided contrastive alignment, which preserves category semantics under low-light conditions.
- On BDD100K Night$\rightarrow$Dawn/Dusk, our framework maintains 39.3% mAP—whereas competing methods suffer significant drops (e.g., AT^19^ drops to 34.0% mAP from 38.4% mAP, AT^19^+CMT^31^ to 36.1% mAP from 39.0% mAP). This advantage of our PoCoMT arises from MT’s stable pseudo-labels, which reduce noise induced by glare/blur, and prototypes that refine misclassified labels for blurred objects (e.g., motion-blurred “cars” are correctly aligned via prototype similarity).

**Table 7:** Ablation study results on Cityscapes $\rightarrow$ FoggyCityscapes and Pascal $\rightarrow$ Watercolor. Best in bold.

		Losses						mAP	
#	Methods	$\mathcal {L}_{mt}$	$\mathcal {L}_{IM}$	$\mathcal {L}_{adv}$	$\mathcal {L}_{ADV}$	$\mathcal {L}_{Intra-PCL}$	$\mathcal {L}_{Inter-PCL}$	Foggy	water
1	PoCoMTw/(✓) & w/o(✗)	✗	✓	✗	✓	✓	✓	45.5	53.2
2		✓	✗	✗	✓	✓	✓	54.2	61.8
3		✓	✓	✓	✗	✓	✓	54.1	61.4
4		✓	✓	✗	✗	✓	✓	53.8	60.2
5		✓	✓	✗	✓	✗	✓	52.6	60.9
6		✓	✓	✗	✓	✓	✗	50.3	59.8
7	PoCoMT	✓	✓	✗	✓	✓	✓	**54.9**	**62.1**
8	PoCoMT w/o ProtoAN							48.6	57.4
9	AT^19^+ ProtoAN							53.6	61.1
10	PT^20^+ ProtoAN							50.0	59.9

### Ablation study

Within this part, extra experimental findings are presented to shed light on where the performance improvement in our proposed approach PoCoMToriginates.

#### Study on crucial loss components

To gauge how various parts of our model contribute, we conduct ablation tests on the tasks Cityscapes $\rightarrow$ FoggyCityscapes (using the “All” subset) and Pascal $\rightarrow$ Watercolor. For a thorough comparison, we single out the influence of six elements within $\mathcal {L}_\mathrm{{PoCoMT}}$ to assess each loss component’s role as outlined in Eq. (1).

Self-training loss $\mathcal {L}_{mt}$: First, we assessed the role of Mean Teacher via ablation, following prior research^34^. This involved removing mutual learning and the Teacher model, then testing the Student model trained cross-domain with only strong augmentation. The first row in Table 7 shows a notable performance decline, indicating that most gains stem from mutual learning using target-domain pseudo labels.

Information maximization loss $\mathcal {L}_{IM}$: Table 7 (second row) reveals that PoCoMT without $\mathcal {L}_{IM}$, which addresses noisy pseudo labels, yielded a slight performance degradation, with -0.7% mAP on Foggycityscapes and -0.40% mAP on Watercolor compared to our PoCoMT. Combining it with other losses produced further improvements.

Adversarial losses $\mathcal {L}_{ADV}$** and **$\mathcal {L}_{adv}$: To explore adversarial learning’s role in PoCoMT, we first removed $\mathcal {L}_{ADV}$ and $\mathcal {L}_{adv}$ from the discriminator, reporting results in Table 7 (fourth row). There are 1.1% and 1.9% mAP drops occurred on Foggycityscapes (smaller real-to-artistic domain gap) and Watercolor (a larger domain gap), respectively.

We also analyzed $\mathcal {L}_{adv}$, adversarial learning without attention mapping (third row in Table 7). Two key observations emerged: minimizing $\mathcal {L}_{adv}$ improved performance, validating the discriminator’s effectiveness; and omitting attention mapping caused minor performance drops due to error spread from noisy pseudo labels. This suggests $\mathcal {L}_{ADV}$’s attention mapping effectively identifies foreground regions, aiding cross-domain knowledge transfer.

Prototype contrastive loss $\mathcal {L}_{pcl}$: when excluded $\mathcal {L}_{pcl}$ from our PoCoMT (eighth row in Table 7), we found 6.3% and 4.7% performance drop on Foggycityscape and Watercolor, respectively. It is evident that tackling both intra-domain and inter-domain biases via prototypical contrastive loss yields positive results. This highlights the importance of our ProtoAN module for modifying the cross-domain MT network’s training pipeline, which reduces noisy pseudo labels from the Teacher model.

Table 7 (the fifth and sixth rows) shows significant performance declines relative to our PoCoMT, confirming $\mathcal {L}_{intra-PCL}$ and $\mathcal {L}_{inter-PCL}$ mitigate intra- and inter-domain biases. Removing $\mathcal {L}_{inter-PCL}$ caused a larger drop than removing $\mathcal {L}_{intra-PCL}$, indicating $\mathcal {L}_{inter-PCL}$ has a stronger influence.

#### Effectiveness of ProtoAN

Table 7 (eighth row) shows removing ProtoAN from PoCoMT reduced mAP to 48.6% on FoggyCityscapes and 57.4% on Watercolor. This validates our method and hypothesis: prototypical contrastive learning in an unbiased space is more effective. Notable performance gains in rows 9 and 10 further confirm ProtoAN’s value in MT self-training.

In the nutshell, our PoCoMT combines ProtoAN and attains the highest mAP values on Cityscapes $\rightarrow$ Foggy-Cityscapes and Pascal $\rightarrow$ Watercolor (refer to the seventh row in Table 7) when compared to PoCoMTw/o ProtoAN (refer to the eighth row in Table 7).

### Further analysis

#### Noise in pseudo-labels

**Fig. 2:**
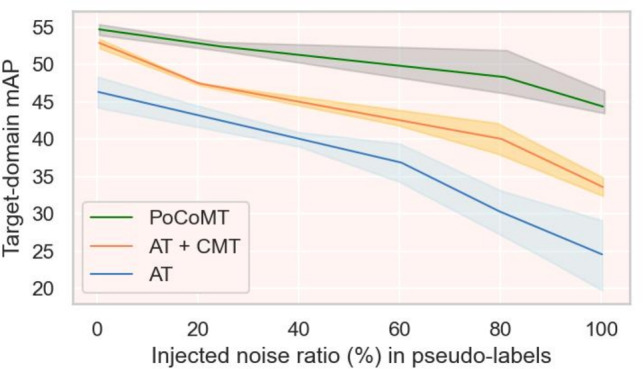
Impact of pseudo-label noise on Foggy Cityscapes target-domain performance. Shadows depict the standard deviation across three runs.

To show that the prototypical contrastive learning in PoCoMT can derive useful learning cues from pseudo-labels even when they contain noise, we devised the following analytical test: during each training step of PoCoMT, we intentionally disrupt the pseudo-labels produced by the Teacher before applying them to the prototypical contrastive loss and detection loss. In particular, for a certain percentage (20% to 100%) of the predicted objects, we assign them random class labels. This injected noise degrades the quality of pseudo-labels, which in turn impairs the domain adaptation process.

Results from this trial are presented in Fig. 2. As more noise is introduced into the pseudo-labels, the performance of all models on the target domain declines gradually and steadily, while the degradation of AT^19^ and its variant AT^19^+CMT^31^ are more pronounced. However, accuracy does not plummet to the level of random guesses, as the models still receives valid guidance from source-domain labels. In comparison, our PoCoMT leverages adaptation-aware prototypical contrastive learning to counteract pseudo-label noise, partially restoring target-domain performance in two ways: First, it reduces performance fluctuations across multiple trials, leading to higher stability when pseudo-labels are noisy. Second, as pseudo-label noise intensifies, our PoCoMT delivers higher performance gains. This trend confirms that our adaptation-aware prototypical contrastive learning can extract valuable information from noisy pseudo-labels to aid unsupervised domain adaptation.

#### Extension to source-free adaptation

Equipped solely with a self-supervised loss for the target domain and a source model pre-trained on the source domain, PoCoMT can be smoothly and easily adapted to source-free UDA-OD (a privacy-sensitive scenario)^22,25,34,103^. In this setup, only unlabeled target data is used to safeguard privacy. Table 8 reveals that PoCoMT attains significant enhancements, highlighting its robustness and scalability. Notably, the performance of PoCoMT both without and with source data is nearly identical across all tasks, including K2C (KITTI $\rightarrow$ Cityscapes), S2C (Sim10k $\rightarrow$ Cityscapes), and C2F (Cityscapes $\rightarrow$ Foggy Cityscapes).

**Table 8:** Results of extension to source-free setting. C2F: Cityscapes $\rightarrow$ Foggy Cityscapes; K2C: KITTI $\rightarrow$ Cityscapes; S2C: Sim10k $\rightarrow$ Cityscapes. Best in bold.

Methods	Source data	mAP		
		C2F	K2C	S2C
Oracle	–	46.3	66.4	66.4
SFOD^103^	✗	33.5	44.6	42.9
BT^24^	✗	39.5	–	–
LPU^26^	✗	38.8	–	–
IRG^25^	✗	37.1	–	–
PETS^104^	✗	35.9	–	–
WSCoL^34^	✗	40.6	–	–
PT^20^	✗	38.7	59.6	54.1
PoCoMT(ours)	✗	**52.8**	**63.2**	**57.7**
PoCoMT(ours)	✓	54.9	64.7	59.5

#### Feature distribution discrepancy of foregrounds

**Fig. 3:**
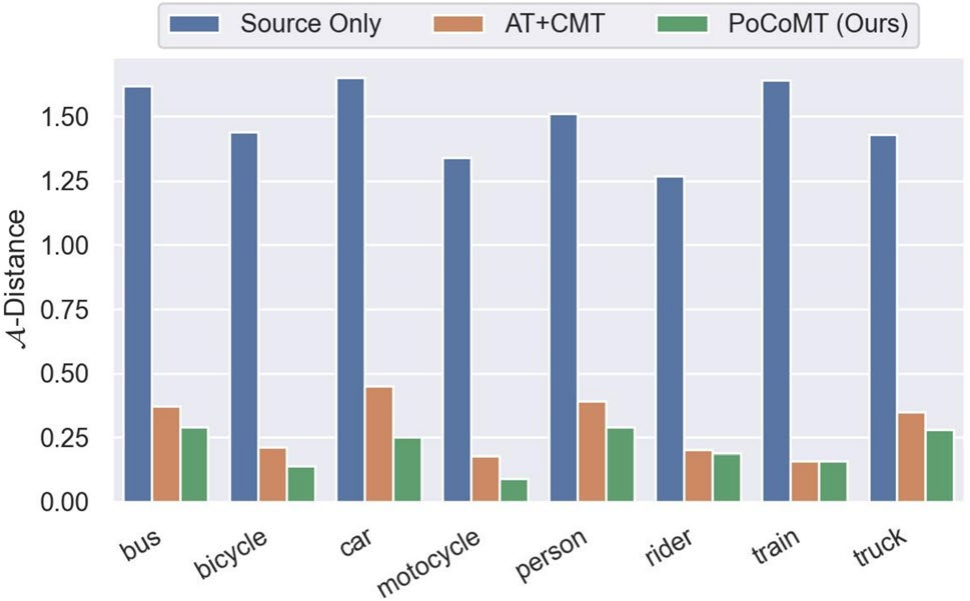
Feature distribution discrepancy of foregrounds.

The theoretical finding in^83^ indicates that $\mathcal {A}$-distance can serve as a measure of domain divergence. In practice, we compute the Proxy $\mathcal {A}$-distance as an approximation, defined as $d_{\mathcal {A}} = 2(1 - \epsilon )$. Here, $\epsilon$ represents the generalization error of a binary classifier^10^ designed to tell apart the domain from which input features originate^11^. Figure 3 shows the distances for each category in the *Adverse Weather Adaptation* task, using foreground features (ground truth) extracted from the *Source Only*, *AT+CMT*, and *Ours* models. In comparison to the non-adaptive model, both *AT+CMT* and *Ours* significantly reduce distances across all categories, underscoring the importance of domain adaptation. Furthermore, because our ProtoAN explicitly optimizes each category’s prototypes, our model achieves a smaller foreground feature distribution gap than the other approaches.

#### Error analysis of highest confident detections

To further confirm the effectiveness of our proposed framework in cross-domain object detection, we examine the errors made by the *Source Only*, *AT+CMT*, and *PoCoMT (Ours)* based on their highest-confidence detections in the *Adverse Weather Adaptation* task. Following the approach in^79^, we classify detections into three error types: (1) Correct (IoU with ground truth $\ge$ 0.5); (2) Mislocalization (0.3 $\le$ IoU with ground truth < 0.5); and (3) Background (IoU with ground truth < 0.3). For each category, we select the top-$N_{gt}$ predictions to analyze error types, where $N_{gt}$ denotes the number of ground truths in that category.

**Fig. 4:**
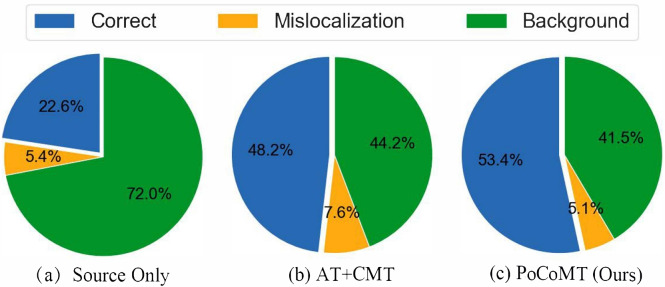
Error analysis of highest confident detections.

The average percentage of each type across all categories is presented in Fig. 4. It is observed that the *Source Only* model tends to misclassify most background regions as false positives (green). In comparison to *AT+CMT*, our model increases the proportion of correct detections (blue) from 48.2% to 53.4% while reducing other error types at the same time. These results demonstrate that the proposed framework can effectively boost true positives and lower false positives, leading to improved detection performance.

#### Qualitative analysis

**Fig. 5:**
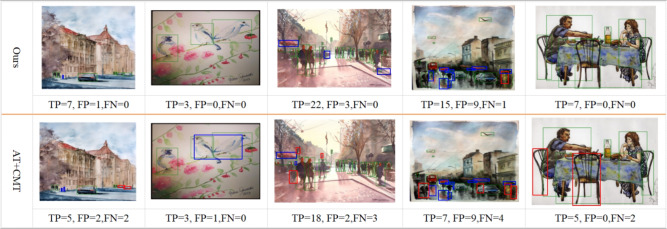
Quantitative results on Pascal $\rightarrow$ Watercolor. Green, blue and red boxes represent true positives (TP), false positives (FP), and false negatives (FN) respectively. *Zoom in for best view.*.

**Fig. 6:**
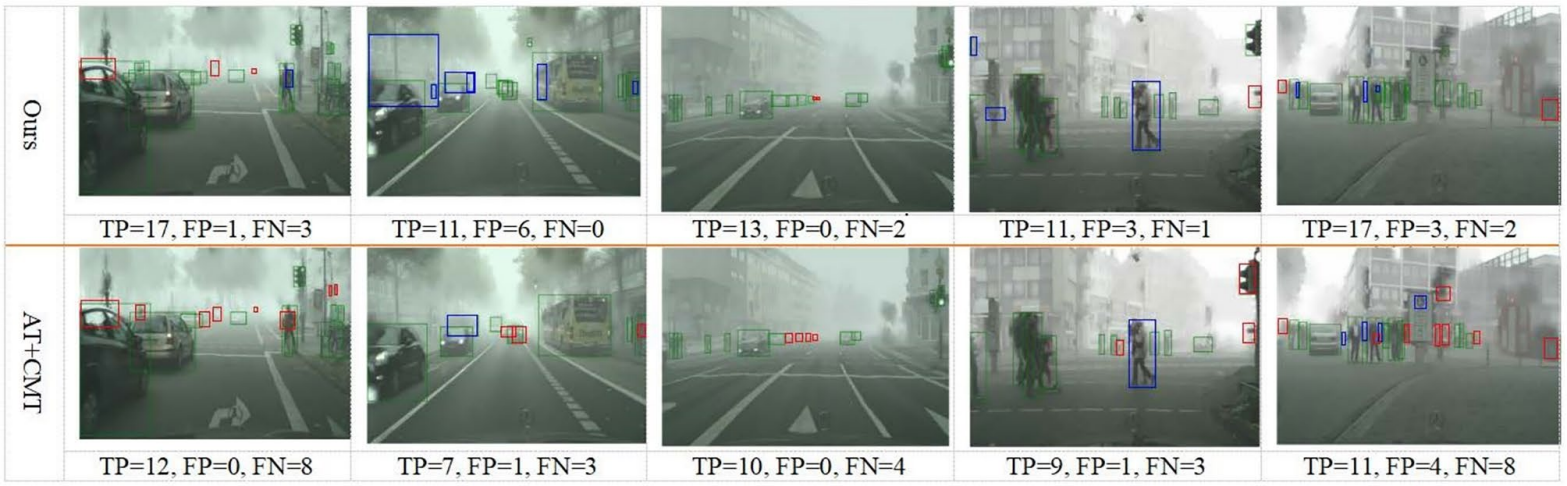
Quantitative results on Cityscapes $\rightarrow$ Foggy Cityscapes. Green, blue and red boxes represent true positives (TP), false positives (FP), and false negatives (FN) respectively. Zoom in for best view..

For qualitative validation of our method, we present detection outcomes for Pascal $\rightarrow$ clipart in Fig. 5 and Cityscapes $\rightarrow$ Foggy Cityscapes in Fig. 6, with AT^19^+CMT^31^ serving as a reference. Clearly, our method detects a greater number of objects without sacrificing precision. In the Cityscapes $\rightarrow$ Foggy Cityscapes task particularly, it can accurately identify objects even when they are heavily hidden by fog. These findings show that our PoCoMT enables detectors to acquire vital category-specific details. From Fig. 5 and Fig. 6, it is evident that the proposed method not only boosts true positives (such as detecting more cars in Fig. 6) but also cuts down on false positives, which aligns with earlier analyses.

#### Analysis of hyper-parameters

**Fig. 7:**
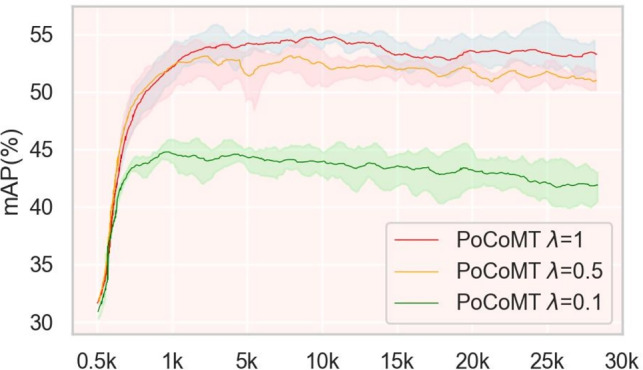
Illustration of training dynamics with different $\lambda$.

**Fig. 8:**
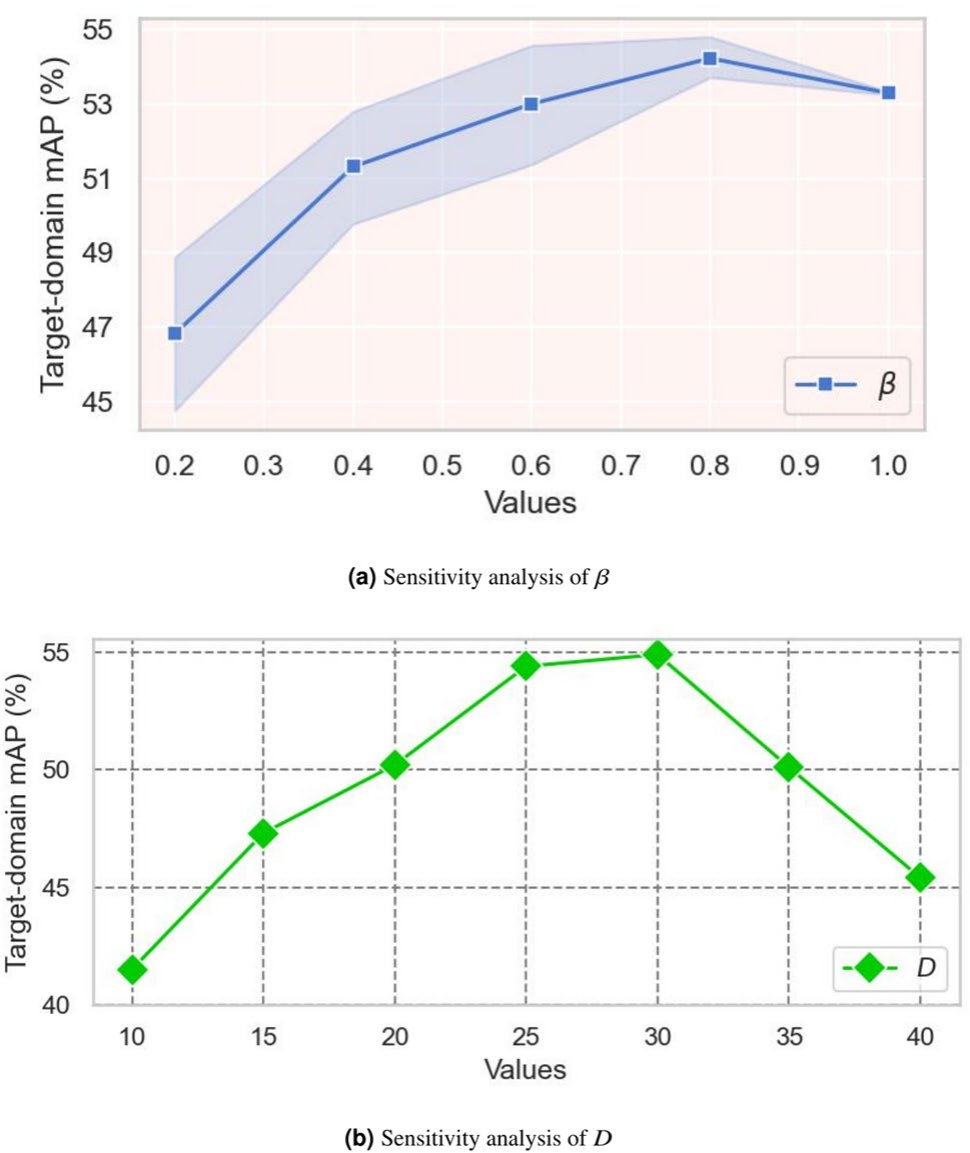
Parameters analysis. (**a**) mAP varying as $\beta$ (top) and (**b**) *D* (bottom) changing.

In this section, we explore how two hyperparameters, $\lambda$ and $\beta$ within the objective function $\mathcal {L}_\mathrm{{PoCoMT}}$, along with the memory bank’s storage length $D$, which affect performance under the Cityscapes $\rightarrow$ FoggyCityscapes adaptation scenario.

First, we assess the role of prototypical contrastive learning in PoCoMT by examining the weight $\lambda$, which balances $\mathcal {L}_{mt-im}$ and $\mathcal {L}_{pcl}$. As depicted in Fig.  7, our model yields relatively stable results as iterations rise. Two key observations emerge from this figure: first, consistent with our expectations, increasing $\lambda$ enhances performance, validating the importance of prototypical contrastive learning in our model; second, omitting the ProtoAN module, viz, $\lambda \rightarrow 0$, causes performance to decline steadily, due to the neglect of semantic structures of domains. This may occur because a high $\lambda$ strengthens semantic knowledge exploration capabilities, while a low $\lambda$ leads to a surge in misclassified samples, introducing excessive noise into the model.

Figure 8a shows that our model maintains stable performance across a broad range of $\beta$ values (0.2$\sim$1.0). Even without $\mathcal {L}_{ADV}$ ($\beta$=0), significant performance improvements can be achieved (see Fig. 8a). This once again proves that our PCL can effectively mitigate the intra/inter-domain biases. What’s more, adaptation performance improves slightly with increasing $\beta$ up to a point, but declines when $\beta$ becomes too large. This indicates that both excessively high and low $\beta$ values harm performance. In our work, this parameter was set without rigorous tuning (all to 0.7$\sim$0.9).

As shown in Fig. 8b, both low and high $D$ values result in decreased mAP. This aligns with our expectations: a small $D$ limits knowledge exploration due to insufficient information, while a large $D$ causes a spike in misclassified samples, flooding the model with excess noise.

## Conclusion

In this work, we investigate the inherent synergies among contrastive learning, prototype learning, and mean-teacher self-training, and propose a novel Prototypical Contrastive Mean Teacher (PoCoMT) framework tailored for UDA-OD. By integrating a custom-designed Prototype Alignment Network (ProtoAN) and an adaptation-aware prototypical contrastive loss, the proposed PoCoMT framework effectively mitigates intra-domain augmentation bias and inter-domain semantic misalignment, two core challenges that hinder the performance of existing UDA-OD methods. The ProtoAN module achieves an optimal balance between feature discriminability, domain invariance, and computational efficiency, rendering it a flexible plug-and-play component compatible with mainstream self-training frameworks. Extensive experimental evaluations across seven benchmark datasets, covering diverse domain shift scenarios, demonstrate that PoCoMT attains state-of-the-art performance.

To enhance the continuity and operability of this research, three targeted future work directions are outlined building on PoCoMT’s core insights and addressing its current limitations. First, we will further optimize ProtoAN via domain-aware neural architecture search (NAS), designing an adaptive structure that adjusts architectural complexity based on domain gap intensity and computational constraints, to achieve optimal performance-efficiency tradeoffs across heterogeneous UDA-OD scenarios. Second, we will integrate dual uncertainty estimation (pseudo-label and prototype uncertainty) with multi-scale and multi-modal feature fusion, refining prototype learning robustness to noisy pseudo-labels and boosting the discriminability of domain-invariant features. Third, we will further extend our PoCoMT to universal domain adaptation scenarios^105–107^, including open-set detection^108^ and multi-source adaptation^102^, broadening its practical applicability to real-world industrial scenarios.

## Data Availability

The data supporting the findings of this study have been properly accounted for and their corresponding access references are explicitly marked within the manuscript. Readers also can directly refer to the following links to download the datasets: The dataset KITTI is available at: http://www.cvlibs.net/datasets/kitti/; The BDD100K is available at: http://bdd-data.berkeley.edu/download.html; The Cityscapes is available at: https://mood.nbpt.edu.cn/cityscapes.zip; The Pascal VOC is available at: https://www.kaggle.com/api/v1/datasets/download/huanghanchina/pascal-voc-2012?dataset_version_number=1; The Foggy-Cityscapes is available at: https://people.ee.ethz.ch/ csakarid/SFSU_synthetic/; The datasets Clipart and Watercolor can be downloaded by respectively executing the following commands: gdown 1LvxwCOfUa-OklIvBJhB8zJlochjJiPFS gdown 1fa2L6oaPSjZ1_WqlTmIp6i2RbdR2y1Pw The dataset Sim10K is available at: https://fcav.engin.umich.edu/sim-dataset/.
